# Rapid determination of medulloblastoma subgroup affiliation with mass spectrometry using a handheld picosecond infrared laser desorption probe†
†Electronic supplementary information (ESI) available. See DOI: 10.1039/c7sc01974b



**DOI:** 10.1039/c7sc01974b

**Published:** 2017-07-21

**Authors:** Michael Woolman, Isabelle Ferry, Claudia M. Kuzan-Fischer, Megan Wu, Jing Zou, Taira Kiyota, Semra Isik, Delaram Dara, Ahmed Aman, Sunit Das, Michael D. Taylor, James T. Rutka, Howard J. Ginsberg, Arash Zarrine-Afsar

**Affiliations:** a Techna Institute for the Advancement of Technology for Health , University Health Network , 100 College Street , Toronto , ON M5G 1P5 , Canada . Email: arash.zarrine.afsar@utoronto.ca; b Department of Medical Biophysics , University of Toronto , 101 College Street , Toronto , ON M5G 1L7 , Canada; c Peter Gilgan Centre for Research and Learning , Hospital for Sick Children , 686 Bay Street , Toronto , ON M5G 0A4 , Canada; d Drug Discovery Program , Ontario Institute for Cancer Research , 661 University Avenue , Toronto , ON M5G 0A3 , Canada; e Department of Surgery , University of Toronto , 149 College Street , Toronto , ON M5T 1P5 , Canada; f Keenan Research Center for Biomedical Science , The Li Ka Shing Knowledge Institute , St. Michael's Hospital , 30 Bond Street , Toronto , ON M5B 1W8 , Canada; g Institute of Biomaterials and Biomedical Engineering , University of Toronto , 164 College Street , Toronto , ON M5S 3G9 , Canada; h Arthur and Sonia Labatt Brain Tumor Research Centre , The Hospital for Sick Children , Toronto , ON M5G 1X8 , Canada; i Developmental & Stem Cell Biology Program , The Hospital for Sick Children , 686 Bay Street , Toronto , ON M5G 0A4 , Canada

## Abstract

Medulloblastoma (MB), the most prevalent malignant childhood brain tumour, consists of at least 4 distinct subgroups each of which possesses a unique survival rate and response to treatment. To rapidly determine MB subgroup affiliation in a manner that would be actionable during surgery, we subjected murine xenograft tumours of two MB subgroups (SHH and Group 3) to Mass Spectrometry (MS) profiling using a handheld Picosecond InfraRed Laser (PIRL) desorption probe and interface developed by our group. This platform provides real time MS profiles of tissue based on laser desorbed lipids and small molecules with only 5–10 seconds of sampling. PIRL-MS analysis of *ex vivo* MB tumours offered a 98% success rate in subgroup determination, observed over 194 PIRL-MS datasets collected from 19 independent tumours (∼10 repetitions each) utilizing 6 different established MB cell lines. Robustness was verified by a 5%-leave-out-and-remodel test. PIRL ablated tissue material was collected on a filter paper and subjected to high resolution LC-MS to provide ion identity assignments for the *m*/*z* values that contribute most to the statistical discrimination between SHH and Group 3 MB. Based on this analysis, rapid classification of MB with PIRL-MS utilizes a variety of fatty acid chains, glycerophosphates, glycerophosphoglycerols and glycerophosphocholines rapidly extracted from the tumours. In this work, we provide evidence that 5–10 seconds of sampling from *ex vivo* MB tissue with PIRL-MS can allow robust tumour subgroup classification, and have identified several biomarker ions responsible for the statistical discrimination of MB Group 3 and the SHH subgroup. The existing PIRL-MS platform used herein offers capabilities for future *in vivo* use.

## Abstract 2

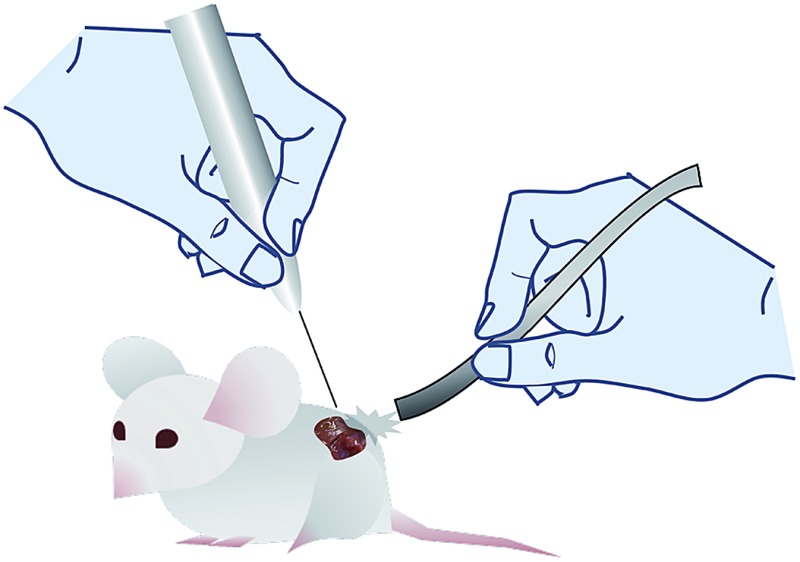
 *In situ* mass spectrometry sampling in the absence of tissue thermal damage.

## Introduction

Medulloblastoma (MB) is a malignant pediatric brain tumour that is comprised of at least 4 distinct molecular subgroups (SHH, WNT, Group 3 and Group 4).^1^ The response to treatment, the prognosis and the overall survival rates are different between MB subgroups. Therefore, molecular subgrouping is *en route* to become part of the risk stratification of MB patients.^2^ With molecular analysis capabilities becoming available at a larger number of clinical sites, molecular subgrouping is already playing an important role in management of patients with gliomas^3^ and is expected to play a pivotal role in the personalized approaches to MB patient care as well. Currently, however, no rapid intraoperative means of determining subgroup affiliation exists to guide extent of resection, thereby minimizing postoperative neurological morbidity. While histopathology and immunohistochemistry methods, along with genomic NanoString DNA analysis and DNA methylation profiling, are used to classify MB subgroups,^4^ intraoperative utility is lacking due to lengthy turnaround times. In the quest to determine MB subgroup affiliation information in a manner that would be actionable during surgery a new analytical platform capable of rapid determination of tumour subgroups must be developed.

Ambient Mass Spectrometry (MS) is a powerful analytical platform capable of resolving the molecular heterogeneity of biological tissues examined under atmospheric conditions.^5–7^ The ambient attribute enables direct *in vivo*, *in situ* or *ex vivo* tissue sampling, often in the absence of extensive sample preparation requirements. The molecular heterogeneity profile of the tissue, also referred to as its MS profile, is comprised of mass to charge (*m*/*z*) ratios of its constituent molecules. This profile can be obtained on timescales suitable for future intraoperative use^6,8^ and is characteristic of each tissue type.^6^ Capitalizing on this notion, experimentally recorded MS profiles can thus be used to identify tissue types. In this quest, rapid tissue identification uses multivariate statistical comparison methods that query the experimentally recorded MS profile of an unknown tissue against those present in a library of validated MS tissue profiles.^5,6^ The multivariate methods are not computationally costly and generally can be performed in a fraction of a second as the MS spectra are acquired. Online model building methods capable of real time MS analysis are reported.^6^

Progressing beyond the tissue differentiation paradigm in distinguishing diseased and healthy tissues, the lipid and small molecule metabolite profiles of biological tissues are shown to have utility in cancer type identification or even tumour subtype determination with many ambient MS methods.^5,6,9–17^ These classes of molecules thus offer superb diagnostic power in determining subtypes of the same cancer based on the specific MS profile of lipids unique to each tumour subtype.^9^ Good concordance with pathology-based classification methods is reported for a variety of human brain tumours^9^ and other cancers.^5,6,8^ Many of these pioneering studies have used Desorption ElectroSpray Ionization Mass Spectrometry (DESI-MS)^18^ where charged microdroplets of a solvent material focused on the surface of a tissue slice or tissue smear^17,19^ bring about extraction, desorption and ionization of tissue lipids and small molecule metabolites. DESI-MS has risen to an era of widespread utility in rapid cancer characterization in the biomedical domain.^5,6^ While a typical DESI-MS scan on the order of ∼1 second is often sufficient to provide robust tissue MS lipid profiles,^19,20^ *in vivo* utility is lacking. The DESI-MS source in its current form cannot be used *in vivo* due to requirements for high electric potential, and the use of solvent materials toxic to the human body. To facilitate intraoperative applications two approaches have been developed. One uses *ex vivo* tissue samples or tissue smears taken to a mass spectrometer located in close proximity to the operating room for off-line analysis, and the other uses real time capture and analysis by MS of the plume of electrocautery widely used in many surgical procedures for online assessment of cancerous tissue *in vivo*.^21^ While electrocautery is thermally destructive and thus cannot be used over healthy tissues due to concerns of damage, residual lipid and small molecule metabolites present in the tumour core survive the diathermy process. These molecules persist in the aerosols generated during diathermy, and can be taken up and desolvated for further online analysis with MS. Tremendous progress has been made in the cancer characterization domain with very high correct tissue classification rates corroborated by gold standard pathology methods.^6^

To further enhance *in vivo* cancer characterization with online MS, it is desirable to have a rapid tissue lipid and small molecule “extraction” method that (1) is efficient, allowing for reduced sample consumption (*i.e.* tissue area to be examined); and (2) minimally damages the tissue surrounding the sampling site, such that the method can be used with fewer reservations in both tumour bed examinations and negative margin assessments *in vivo*. The current implementation of the electrocautery based MS methods^21^ requires *a priori* determination of the cancerous region using surgeon's input or other image modality data to provide an avoidance mechanism for healthy tissue, and is a valuable tool for *in vivo* tumour grading. In this quest, the Rapid Evaporative Ionization Mass Spectrometry (REIMS) interface, developed initially for the analysis of the plume of electrocautery,^22^ has been shown to be compatible with a variety of tissue aerosolization methods, including ultraviolet (UV) and infrared (IR) laser desorption,^23^ and ultrasonic aspiration.^24^

Recently, the Picosecond InfraRed Laser (PIRL) has been shown to rapidly “extract” *via* a desorptive mechanism,^25^ in the absence of significant thermal damage,^26^ tissue molecular content in the form of a gas phase plume^27^ expanding rapidly in the atmosphere.^28^ Subsequent capture and analysis by mass spectrometry of this plume has been demonstrated to be feasible upon coupling to an appropriate post desorption ionization source for MS imaging applications.^27^ Tissue desorption with a picosecond IR pulse is a highly efficient process due to the strong coupling between libration and vibrational modes of water on this timescale.^29^ The bulk of the impulsive energy deposited into the vibrational mode of tissue water molecules is converted into desorption, liberating water and tissue constituent molecules and ejecting them to the gas phase in the absence of significant thermal damage to the tissue.^26^ Capitalizing on the highly efficient nature of the laser processing with PIRL,^29^ that even allows cutting of bone material^30^ which has low water content compared to soft tissue, we hypothesized that lipid species may be expected in the laser desorption plume. Based on this assumption, we recently demonstrated online coupling between PIRL desorption and MS for real time diagnostic applications through use of a 2 m long flexible collection tube coupled to a modified heated inlet capillary of a Time of Flight (TOF) MS instrument, capable of resolving transient signals typical to laser desorption mass spectrometry methods. The heated inlet promotes thermal desolvation of the laser desorbed, negatively charged tissue lipids,^31^ condensed and possibly re-solvated during the rapid cooling and plume expansion stage of the PIRL desorption process under atmospheric conditions.^28^ This laboratory built interface was shown to allow real time tissue profiling with *in situ* sampling in 5–10 seconds of total data collection, followed by post collection data analysis and statistical treatment,^31^ and adds to the current methods of laser desorption ionization (LDI) mass spectrometry.

In this work, we used 19 independent subcutaneous murine xenograft tumours from 6 different established human MB cell lines belonging to MB subgroups of Sonic Hedgehog (SHH) and Group 3. A successful MB subgroup affiliation (98% accuracy) was achieved using PIRL-MS analysis with 5–10 seconds of sampling, assessed through supervised multivariate statistical analysis, utilizing close to 200 data points, with robustness confirmed with an iterative 5%-leave-out-and-remodel test. Additional high resolution LC-MS study of the captured laser desorption plumes allowed identification of *m*/*z* values that contributed the most to the statistical discrimination of PIRL-MS profiles of MB subgroup tumours. In anticipation of potential future clinical utility, a detailed discussion of analytical performance, origin of the outlier data points, and the duty cycle is also presented in the ESI.† We thus provide a proof-of-principle demonstration of the utility of the online PIRL-MS setup previously developed by our group^31^ in rapid determination of MB subgroup affiliation. The small lipid and metabolite profiles for MB reported here in this orthogonal study will add to the existing knowledge of protein and ganglioside biomarkers identified by other mass spectrometry methods including LC-MS/MS and Matrix Assisted Laser Desorption Ionization Mass Spectrometry (MALDI-MS).^32,33^

## Experimental methods

### MB murine xenograft tumours

All cells were cultured at 37 °C and 5% CO_2_. Human medulloblastoma cell lines were grown in media containing various concentrations of amino acids, salts, vitamins and between 10–20% Fetal Bovine Serum (FBS) (Wisent Inc., St. Bruno, QC, Canada). All animal procedures were approved by the Animal Care Committee at the Toronto Centre for Phenogenomics (TCP). Animal-use-protocols are in accordance with the guidelines established by the Canadian Council on Animal Care and the Animals for Research Act of Ontario, Canada. Under isoflurane anesthesia, mice were injected with 2.5 million cells into both flank regions, total injection volume was 100–200 μL into each flank. After tumour volume had reached 500–800 mm^3^ or 5 weeks post injection, the mice were euthanized and the tumours were resected for MS analysis. 19 tumours were used for PIRL-MS with the break down by cell line as follows: D341, *n* = 4; D458, *n* = 3; MED8A, *n* = 2; DAOY, *n* = 3; ONS76, *n* = 3; UW228, *n* = 4.

### PIRL MS analysis

The handheld PIRL-MS source^31^ using a PIRL 3000 unit (Attodyne Lasers, currently Light Matter Interactions) is used as described previously with a 2 m long Tygon tube connected to the heated inlet (150 °C) capillary of a DESI-MS collection source (Waters).^31^ The laser fiber tip (500 μm spot, 3000 ± 100 nm, 300 ± 100 ps at 1 kHz, fluence of ∼0.15 J cm^–2^), was rastered over a ∼1–5 mm^2^ area for 5–10 seconds without touching the specimen, with the tip of the plume collection tube 1–2 mm away from the site of desorption. PIRL-MS spectra (from *m*/*z* 100 to *m*/*z* 1000) were collected on a Xevo G2XS Quadrupole-Time-Of-Flight Mass Spectrometer (Q-TOF-MS, Waters) in the negative ion mode. Additional details of laser desorption parameters and the setup are previously reported.^31^ For MB sample analysis, subcutaneous xenograft tumours were surgically exposed, harvested and subjected to PIRL-MS sampling with data collection times not exceeding 10 seconds. Each tumour was sampled at least 10 times from different regions both on the surface and from its core (tumours were halved) to capture spatial heterogeneities akin to those present in real world samples. A grand dataset of 194 PIRL-MS data points (*i.e.* spectra) collected over 5–10 seconds of PIRL-MS sampling was generated.

### Data analysis

The 194 data files were divided into two folders, one for Group 3 and one for SHH, and submitted to MetaboAnalyst for Partial Least Squares Discriminant Analysis (PLS-DA). Details of Metaboanalyst settings are as reported previously^31^ with 1 notable exception: mass tolerance was set to 100 mDa due to the lack of correction for mass shift. In cases where a 25 mDa tolerance was used, the spectra were corrected using the accurate mass of 717.5076 (Table 1). While this peak was more intense in Group 3 samples, it was present in all samples at levels well above the background.

**Table 1:** LC-MS analysis of the laser desorption plume of MED8A and DAOY tumours captured on a filter paper. The *m*/*z* values from direct analysis of MED8A and DAOY tumours (Fig. 1) are listed in the left column. These values were used to guide targeted analysis of the plume captured on filter paper with LC-MS. The observed and theoretical mass values, along with mass difference in both mDa and ppm are provided. Molecular formulas are based on accurate mass values and isotopic pattern, and possible hits from LipidMaps database are provided. Since only MED8A and DAOY tumours were used, the last column indicates whether changes in the relative abundance of these *m*/*z* values were statistically significant over all 6 cell line data reported in Fig. S4 (PLA-SDA box plots of the multivariate analysis shown in Fig. 2). Note that the LC-MS method used cannot resolve the isomeric possibilities

MED8A *m*/*z* direct PIRL MS	LC-MS analysis of captured PIRL desorption plume							Statistically significant change in relative abundance over 6 cell line data
	Observed *m*/*z*	Theoretical *m*/*z*	Shift mDa	Shift ppm	Molecular formula	Type of ion	Lipid map hit(s)	
134.05	134.0471	134.0467	0.4	3	C_5_H_4_N_5_	N/A	N/A	Yes
255.25	255.2321	255.2324	0.3	–1.2	C_16_H_31_O_2_	[M – H]^–^	FA(16 : 0)	No
281.25	281.2483	281.2481	0.2	0.7	C_18_H_33_O_2_	[M – H]^–^	FA(18 : 1)	Yes
303.23	303.2325	303.2324	0.1	0.3	C_20_H_31_O_2_	[M – H]^–^	FA(20 : 4)	No
305.25	305.2478	305.2481	–0.3	–1	C_20_H_33_O_2_	[M – H]^–^	FA(20 : 3)	Yes
329.25	329.248	329.2481	–0.1	–0.3	C_22_H_33_O_2_	[M – H]^–^	FA(22 : 5)	Yes
391.25	391.2251	391.225	0.1	0.3	C_19_H_36_O_6_P	N/A	N/A	No
417.25	417.2364	417.2366	–0.2	–0.5	C_16_H_38_N_2_O_8_P	N/A	N/A	Yes
572.50	572.4808	572.4809	–0.1	–0.2	C_34_H_67_NO_3_Cl	[M + Cl]^–^	Cer(d34 : 1)	Yes
629.50	629.4902	629.4912	–1	–1.6	C_37_H_70_O_5_Cl	[M + Cl]^–^	DG(34 : 1)	No
659.50	659.4647	659.4652	–0.5	–0.8	C_36_H_68_O_8_P	[M – H]^–^	PA(33 : 1)	Yes
						[M – H]^–^	PA(O-33 : 2(OH))	
						[M – H]^–^	PA(P-33 : 1(OH))	
663.50	—	—	—	—	—	—	—	Yes
687.55	687.4969	687.4965	0.4	0.6	C_38_H_72_O_8_P	[M – H]^–^	PA(35 : 1)	Yes
						[M – H]^–^	PA(O-35 : 2(OH))	
						[M – H]^–^	PA(P-35 : 1(OH))	
713.55	713.5112	713.5121	–0.9	–1.3	C_40_H_74_O_8_P	[M – H]^–^	PA(37 : 2)	No
						[M – H]^–^	PA(P-37 : 2(OH))	
717.55	717.5067	717.507	–0.3	–0.4	C_39_H_74_O_9_P	[M – H]^–^	PG(O-33 : 2)	No
						[M – H]^–^	PG(P-33 : 1)	
						[M – H]^–^	PA(36 : 1(OH))	
744.58	744.554	744.5543	–0.3	–0.4	C_41_H_79_NO_8_P	[M – H]^–^	PC(33 : 1)	No
						[M – H]^–^	PE(36 : 1)	
						[M – H]^–^	PC(O-33 : 2(OH))	
						[M – H]^–^	PC(P-33 : 1(OH))	
						[M – H]^–^	PE(O-36 : 2(OH))	
						[M – H]^–^	PE(P-36 : 1(OH))	
875.80	—	—	—	—	—	—	—	Yes

DAOY *m*/*z* direct PIRL MS	LC-MS analysis of captured PIRL desorption plume							Statistically significant change in relative abundance over 6 cell line data
	Observed *m*/*z*	Theoretical *m*/*z*	Shift mDa	Shift ppm	Molecular formula	Type of ion	Lipid map hit(s)	
327.25	327.2323	327.2324	–0.1	–0.3	C_22_H_31_O_2_	[M – H]^–^	FA(22 : 6)	No
691.52	691.491	691.4914	–0.4	–0.6	C_37_H_72_O_9_P	[M – H]^–^	PA(34 : 0(OH))	Yes
						[M – H]^–^	PG(O-31 : 1)	
						[M – H]^–^	PG(P-31 : 0)	
709.51	709.4809	709.4808	0.1	0.1	C_40_H_70_O_8_P	[M – H]^–^	PA(37 : 4)	Yes
710.51	—	—	—	—	—	—	—	Yes
721.55	721.5166	721.5172	–0.6	–0.8	C_42_H_74_O_7_P	N/A	N/A	Yes
723.53	—	—	—	—	—	—	—	Yes
733.52	—	—	—	—	—	—	—	Yes
737.55	737.5117	737.5121	–0.4	–0.5	C_42_H_74_O_8_P	[M – H]^–^	PA(39 : 4)	Yes
739.53	739.4910	739.4914	–0.4	–0.5	C_41_H_72_O_9_P	[M – H]^–^	PA(38 : 4(OH))	Yes
743.55	743.5225	743.5227	–0.2	–0.3	C_41_H_76_O_9_P	[M – H]^–^	PA(38 : 2(OH))	Yes
							PG(P-35 : 2)	
751.55	—	—	—	—	—	—	—	Yes
765.55	765.5064	765.5070	–0.6	–0.8	C_43_H_74_O_9_P	[M – H]^–^	PA(40 : 5(OH))	Yes
766.56	766.5386	766.5387	–0.1	–0.1	C_43_H_77_NO_8_P	[M – H]^–^	PC(35 : 4)	Yes
						[M – H]^–^	PE(38 : 4)	
						[M – H]^–^	PE(O-38 : 5(OH))	
						[M – H]^–^	PE(O-38 : 5(OH))	
						[M – H]^–^	PE(P-38 : 4(OH))	
767.55	767.5222	767.5227	–0.5	–0.7	C_43_H_76_NO_9_P	[M – H]^–^	PA(40 : 4(OH))	Yes
770.59	770.5693	770.5700	–0.7	–0.9	C_43_H_81_NO_8_P	[M – H]^–^	PC(35 : 2)	No
						[M – H]^–^	PC(P-35 : 2(OH))	
						[M – H]^–^	PE(38 : 2)	
						[M – H]^–^	PE(O-38 : 3(OH))	
						[M – H]^–^	PE(P-38 : 2(OH))	
794.60	—	—	—	—	—	—	—	Yes

### LC-MS analysis of the captured PIRL desorption plume

The plume of PIRL desorption was collected on a cellulose filter paper (Whatman) placed in vacuum line of a suction unit pump (Laerdal) providing a maximum of 500 mmHg vacuum as depicted in Fig. S3.† The filter paper, cut to the diameter of ∼0.7 cm, stored at –80 °C in a microfuge tube and was left on ice for 5 min, and then was transferred to a new glass vial. Approximately 1 mL of chloroform was added to the vial and vigorously mixed by vortexing for 30 s. The solution was transferred to a new glass vial and evaporated to dry under nitrogen flow. The lipid extract was reconstituted with methanol/chloroform (1/1, v/v, 200 μL) prior to analyses. A blank filter paper was used to prepare the blank solution.

The chromatographic separation of samples (injection volume of 3 μL, kept at 10 °C) for LC-MS analysis (negative ion mode, *m*/*z* 50–1200) was performed on an ACQUITY UPLC I-Class system (Waters) coupled to a Synapt G2-S Q-TOF-MS (Waters) equipped with a LockSpray dual electrospray ion source operating with the following source parameters; capillary voltage of 0.8 kV, cone voltage of 25 V, source temperature of 150 °C, desolvation temperature of 500 °C, cone gas flow of 150 L h^–1^, desolvation gas flow of 600 L h^–1^. The separation of fatty acids used an ACQUITY UPLC HSS-T3 column (2.1 × 50 mm, 1.8 μm, Waters; at 40 °C), and phospholipids were separated using an ACQUITY UPLC protein C4 column (2.1 × 50 mm, 1.7 μm, Waters; at 55 °C). A gradient (using flow-rate of 0.4 mL min^–1^) was established with water/acetonitrile (2/3, v/v) as phase A, and acetonitrile as phase B. A linear gradient with 100–0% phase A (0–18 min), followed by holding at 100% B for 0.1 min was applied. Then, a gradient back to 100% phase A (18.1–19.0 min) was used (held for 1.0 min for re-equilibration).

Accurate mass measurements utilized the LockSpray automated exact mass measurement mode of the instrument. Two [M – H]^–^ ions of *m*/*z* 236.1035 and 554.2615 of the Leucine-Enkephalin compound were used as reference for lock-spray, with the following configurations; frequency of 15 s, cone voltage of 25 V, collision energy of 40 V. Data acquisition was performed on MassLynx 4.1 (Waters), and accurate mass information was submitted to LipidMaps database^34^ for the identification of lipid compounds.

## Results and discussion

### MB classification with PIRL-MS sampling

To examine the potential utility in the determination of MB subgroup affiliation with 5–10 seconds of tissue sampling with the handheld PIRL-MS analysis probe recently reported by our group,^31^ we prepared subcutaneous murine xenograft tumours belonging to two MB subgroups (Sonic Hedgehog (SHH) and Group 3) for which multiple established human cell lines existed. We then subjected *ex vivo* tumour samples thereof to PIRL-MS data analysis.

A drawback with xenograft studies is that a murine model prepared from a single established cancer cell line does not capture the heterogeneity seen in tumours from a patient population. It is thus important to ensure subgroup classification using PIRL-MS is not hampered by the intrinsic biological heterogeneity of tumour samples. To address this caveat to some extent, we used xenograft tumours from 6 different established MB cell lines: D341, D458, MED8A (for Group 3) and ONS76, DAOY, UW228 (for the SHH subgroup). We then combined the PIRL-MS data of these tumours into their respective MB subgroups such that some level of intrinsic biological heterogeneity, albeit to a lesser extent than expected from patient samples, is captured in our analysis.

**Fig. 1:**
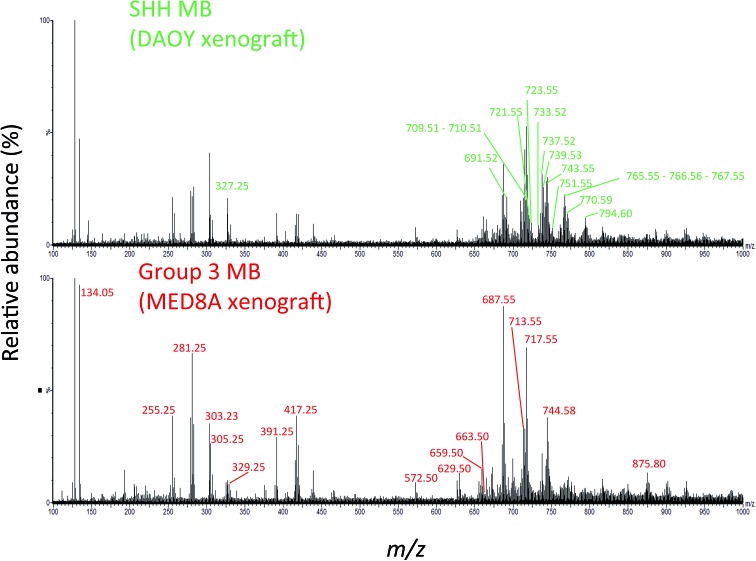
The PIRL-MS spectra of SHH and Group 3 MB tumours. We used DAOY and MED8A derived xenografts for this assessment as representatives for the SHH and Group 3 MB, respectively. These two particular tumours were chosen only on the basis of sample availability. The PIRL-MS spectra were collected for 10 seconds in the negative ion mode using the interface described previously.^31^ The differentiating *m*/*z* values in each of the spectra are labeled. As can be seen, the specificity of PIRL extracted lipids results in different PIRL-MS lipid profiles for MB subgroups. Table 1 provides a list of the *m*/*z* ratios characteristic to each MB subgroup.

We hypothesized that laser desorbed molecules present in the *m*/*z* 100–1000 range of the 194 PIRL-MS spectra recorded (5–10 seconds of laser desorption sampling per spectrum) may provide subgroup-specific MS profiles that could be used to distinguish Group 3 MB from its SHH counterpart. Fig. 1 shows representative PIRL-MS spectra for both Group 3 MB, as represented by a MED8A xenograft tumour, and for the SHH subgroup, as exemplified by xenograft tumour prepared from the DAOY cell line. These spectra were collected with 5–10 seconds of sampling and contain unique, subgroup-specific *m*/*z* values, as highlighted. The PIRL-MS spectra of Group 3 and SHH MB are significantly different from each other, attesting to the specificity of laser desorption of tissue lipids with PIRL. Fig. S1† illustrates the schematics of the experimental setup used for *ex vivo* tissue analysis with PIRL-MS.

**Fig. 2:**
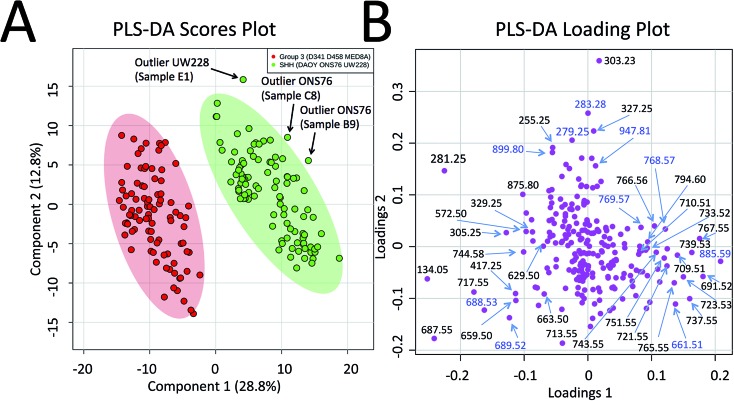
Statistical discrimination of the SHH and Group 3 MB based on 5–10 second PIRL-MS analysis. PIRL-MS spectra collected in the negative ion mode in less than 10 seconds from 19 tumours comprised of murine xenografts from 6 established MB cell lines of D341, D458 and MED8A (for Group 3) and DAOY, ONS76 and UW228 (for SHH) with 10 repetitions from each tumour were processed as described and subjected to multivariate analysis using PLS-DA through the MetaboAnalyst portal. (A) PLS-DA scores plot. 194 data points, each comprised of a single 5–10 second PIRL-MS spectrum, are shown to be statistically grouped into their expected classes. The shaded ovals represent the 95% confidence interval. No data point from one class is found within the 95% confidence interval area of the other class (misclassification). Three outliers were detected outside the 95% confidence interval and are discussed in text. Internal sample name designation names are used. (B) The PLS-DA loading plot. The data points represent individual *m*/*z* values in the rank order that they contribute to the statistical discrimination between PIRL-MS profiles of the SHH and Group 3 MB tumours shown in the scores plot in (A). The *m*/*z* values that are located to the periphery (left and right), along the axis of separation shown in (A) of the loading plot space contribute most strongly to the statistical discrimination of the two MB subgroups examined in this work, and could be considered as univariate biomarker ions of each MB subgroup. The majority of the *m*/*z* values identified in PLS-DA loading plot were present in the single cell line representative PIRL-MS spectra shown in Fig. 1 using DAOY and MED8A tumours. The *m*/*z* values labeled in blue font are identified through PLS-DA but were not typical to MED8A or DAOY profiles that constituted the basis of our targeted assessment.

Progressing beyond single MED8A and DAOY tumours as representatives of Group 3 and SHH MB, we grouped the collective PIRL-MS data from all 6 cell lines listed above into their respective MB subgroups. The grand dataset of 194 PIRL-MS spectra was then subjected to the supervised multivariate method of Partial Least Squares Discriminant Analysis (PLS-DA)^35^ to assess the success rate of MB subgroup affiliation determination with 5–10 seconds of PIRL-MS sampling. Fig. 2A shows the PLS-DA scores plot that clearly demonstrates the statistical discrimination between PIRL-MS data points belonging to two MB subgroups examined. Since each data point is collected with only 5–10 seconds of sampling the determination of subgroup affiliation achieved herein is considerably faster than the competing methods of immunohistochemistry and NanoString DNA sequencing. No overlap with the 95% confidence interval area (shaded ovals) between SHH and Group 3 data is seen over this large dataset. While a few outliers (*n* = 3) locate to the outside of the 95% confidence interval boundaries, no misclassified data points are present. Misclassification is defined as a data point from one subgroup presenting itself within the 95% confidence interval of the other group. For these comparisons and throughout this manuscript, data points that localized within the 95% confidence interval border of a subgroup were considered as belonging to that subgroup. The success rate for correct MB subgroup affiliation prediction, defined as the percentage of PIRL-MS spectral data points that are correctly classified into the 95% confidence interval of their expected MB subgroup, was 98%. Therefore, PIRL-MS spectra collected in only 5–10 seconds, in the absence of additional averaging, are sufficient to provide predictable MB subgroup classification statistics. In the anticipation of potential future clinical applicability, additional discussions around outlier data points, duty cycle and reproducibility of PIRL-MS measurements have been included in the ESI† accompanying this manuscript. Below we will discuss the statistical robustness of our observations.

### Statistical validity of MB classification

Since prior knowledge of the expected subgroup affiliation existed for all of the MB tumours examined here, unsupervised multivariate statistical methods such as Principal Component Analysis (PCA) were not pursued to discover latent features present in the PIRL-MS spectra.^35^ While PCA can also be used to reveal group affiliations, its application for this purpose requires within group variations that are less than between group variations.^35^ Considering group affiliation information existed for our samples, and the extent of within group variation was not available to justify use of PCA we chose PLS-DA as recommended.^35^ However, to address the statistical robustness of the separation seen in Fig. 2A we performed a 5% leave-out-and-remodel test. We iteratively removed 5% of the PIRL-MS data points from both SHH and Group 3 datasets, and considered the 5% data points as pseudo-unknown entities. We then created a model based on the 95% remainder of all data points, and performed a 3 component PLS-DA analysis where the two reference datasets consisted of the SHH and Group 3 PIRL-MS data (95%, as model), with the test dataset being the 5% pseudo-unknowns. The PIRL-MS data points of the pseudo-unknowns were then ranked for how they grouped within the 95% interval area of the expected MB subgroup based on the iterative model predictions. Fig. S2† shows the resultant 21 PLS-DA scores plots for pseudo-unknown datasets that were iteratively left out and scored for expected MB grouping. Here we oversampled the dataset for an additional 7% to create identical weight of representation for both SHH and Group 3 data points. Based on the 21 runs of the iterative 3-way PLS-DA comparisons performed (210 oversampled datasets), a success rate for correct MB affiliation prediction of 94% was achieved. The results consisted of 12 outliers (Fig. S2†). No misclassified data points were seen, and none of the 21 models showed overlap in 95% confidence interval indicative of the failure of the model.

### Identification of MB subgroup biomarker ions

To further highlight the individual *m*/*z* values (or biomarker ions) that best characterize MB SHH and Group 3 cancers, in Fig. 2B we show the PLS-DA loading plots. This representation illustrates how individual *m*/*z* values contribute to the statistical discrimination between MB Group 3 and the SHH subgroup shown in Fig. 2A. The *m*/*z* values that are located at the periphery of the plot (*i.e.* along the axis of the statistical separation between Group 3 and SHH MB) contribute most strongly to the discrimination between the two MB subgroups. The loading plots, thus, provide a pictorial representation of the rank order with which univariate *m*/*z* values contribute to the statistical discrimination visualized by the multivariate PLS-DA scores plot shown in Fig. 2A.

To identify the *m*/*z* values that were used to distinguish MB SHH and Group 3 samples we captured the plume of laser desorption from representative MED8A (for Group 3) and DAOY (for SHH subgroup) tumours on a piece of filter paper and performed high resolution MS analysis on the PIRL extracted lipids captured on said paper using Liquid Chromatography (LC) hyphenated with MS analysis (LC-MS). In this work we thus used PIRL-MS for the identification of *m*/*z* values that contribute to the statistical discrimination of two MB subgroups, and high resolution LC-MS to assign molecular identity to said *m*/*z* values. The assignment of identity to MB subgroup discriminating ions enables future investigation of how metabolic pathways intersect with cancer biology in MB pathology. Similar studies on other cancers have been undertaken that signify the importance of metabolic investigations in advancing our fundamental knowledge of cancer biology, and how metabolic and genetic changes relate to each other in the context of pathologic pathways.^15,36^

Fig. S3† shows the schematics of the plume capture system used that consisted of a filter paper placed in the vacuum line flanked by a funnel at the proximal end (desorption site) and a commercial surgical aspirator vacuum pump at the distal end. To maximize the MS signal, the collection continued for ∼5 min until the plume material visibly stained the filter paper. We then subjected the filter paper to extraction of lipids with chloroform and subjected said extract to high resolution LC-MS analysis. Since the laser plumes were captured from 2 representative SHH and Group 3 tumours only, we did not use the PLS-DA loading plot results (Fig. 2B) that are based on 6 cell line results to guide the selection of ions of interest for this analysis. Instead, we picked *m*/*z* values found to differentiate the representative DAOY and MED8A PIRL-MS spectra (Fig. 1) to perform our targeted LC-MS analysis. While the PIRL-MS spectra of MED8A and DAOY tumours shown in Fig. 1 contained the majority of the *m*/*z* values known to distinguish Group 3 MB from the SHH subgroup by multivariate analysis (see Fig. 2B, PLS-DA loading plot), there may be additional putative *m*/*z* biomarker ions for Group 3 and SHH subgroups for which we have not pursued targeted identification, as these ions did not present themselves in the PIRL-MS spectra of the captured DAOY and MED8A plume samples. These *m*/*z* values are highlighted in blue font in the PLS-DA loading plot (Fig. 2B). As suggested by the PLS-DA Box plots that show how the relative abundance of these *m*/*z* values change between Group 3 and the SHH subgroup (Fig. S4†), some (but not all) of these ions exhibited significant changes in intensity between Group 3 and SHH subgroups and could be further investigated using collection and targeted analysis of the plume of PIRL desorption of MB tumours from cell lines other than MED8A and DAOY analyzed herein. In Table 1, however, we have further indicated, for each *m*/*z* value seen in the PIRL-MS spectra of DAOY and MED8A tumours, whether they, based on data compiled from all 6 MB cell lines represented in the Box plots of Fig. S4,† exhibit a statistically significant change in relative abundance between Group 3 and SHH tumours. While opportunities to extend the targeted analysis to all *m*/*z* values shown in the PLS-DA loading plot (Fig. 2B) exist, this was not pursued further as we intend to undertake such an assessment using human samples.

Out of the 17 laser desorbed Group 3 *m*/*z* values listed in Table 1 that contributed to the statistical discrimination of the PIRL-MS spectra of MED8A from DAOY tumours, 15 *m*/*z* values were present in the LC-MS spectra of the liquid extract prepared from the filter paper that captured the laser plume. Of these 15 hits, 12 *m*/*z* values were identified, further corroborated with hits present in the LipidMaps online tool.^34^ For the other three, only putative molecular formulas are presented in Table 1 based on accurate mass information. These compounds were not present in the lipid map database. As shown in Table 1, PIRL desorption releases a plethora of fatty acid (FA) chains such as FA(16 : 0), FA(18 : 1), FA(20 : 4), FA(20 : 3) and FA(22 : 5), diacylglycerol DG(34 : 1), phosphatidic acids such as PA(33 : 1), PA(35 : 1) and PA(37 : 2) as well as fragments of phospholipids such as phosphatidylcholines PC(33 : 1) or potential phosphatidylethanolamines to the gas phase. The classification is as reported.^37^ A comparison between MS spectra of lipid extract directly prepared from tumour tissue and that of the captured plume reported here is required to answer the intriguing question of whether it is the PIRL desorption process that results in fragmentation of lipids. This is beyond the scope of this proof-of-principle demonstration and will be conducted in the future. It is an interesting question to answer because any concomitant molecular fragmentation induced by PIRL desorption in lipid molecules is bound to produce MS spectra reminiscent of a conventional MS/MS analysis using collision induced dissociation methods that could enhance the specificity of PIRL based diagnostics using profiling methods. Multivariate separation using MS/MS data is analytically more powerful and specific than profiling based on the MS data alone. To this end, a systematic analysis using standard lipid compounds is required to map out the dependence of any potential fragmentation on the fluence of the laser.

In Table 1 we also list the identity assignments for SHH biomarker ions represented by high resolution MS analysis done on the captured plume of a DAOY tumour. Here, out of the 16 *m*/*z* values present in the PIRL-MS spectra of this tumour, we were able to identify 11 with our targeted approach. These *m*/*z* values were shown in the PLS-DA loading plot (Fig. 2B) to contribute to statistical discrimination of MB tumours based on combined data from more than one cell line. Overall, lipids characterizing SHH MB based on DAOY results (Table 1) were similar in nature to those listed above for Group 3, albeit with fewer fatty acid chains seen in the spectra. While the desorption conditions remained the same between MED8A and DAOY samples, fewer fatty acid chains were observed there.

It is important to note that the chemical composition of an MS profile heavily depends on the nature of the extraction, desorption and the ionization methods used. The commonality we observed between direct PIRL-MS and LC-MS profiles may stem from the study design that used the plume of laser desorption for LC-MS. To further validate our findings, and to provide a comprehensive overview of metabolic changes that accompany MB pathology, orthogonal investigation of MB biomarker ions using other MS methods may be necessary. To this end, preliminary unpublished observations from our laboratory using the same tumour samples repurposed for Desorption Electrospray Ionization Mass Spectrometry (DESI-MS) is shown to recover some *m*/*z* values also seen with PIRL-MS.

### Molecular classification of MB cell lines based on PIRL-MS profiling

**Fig. 3:**
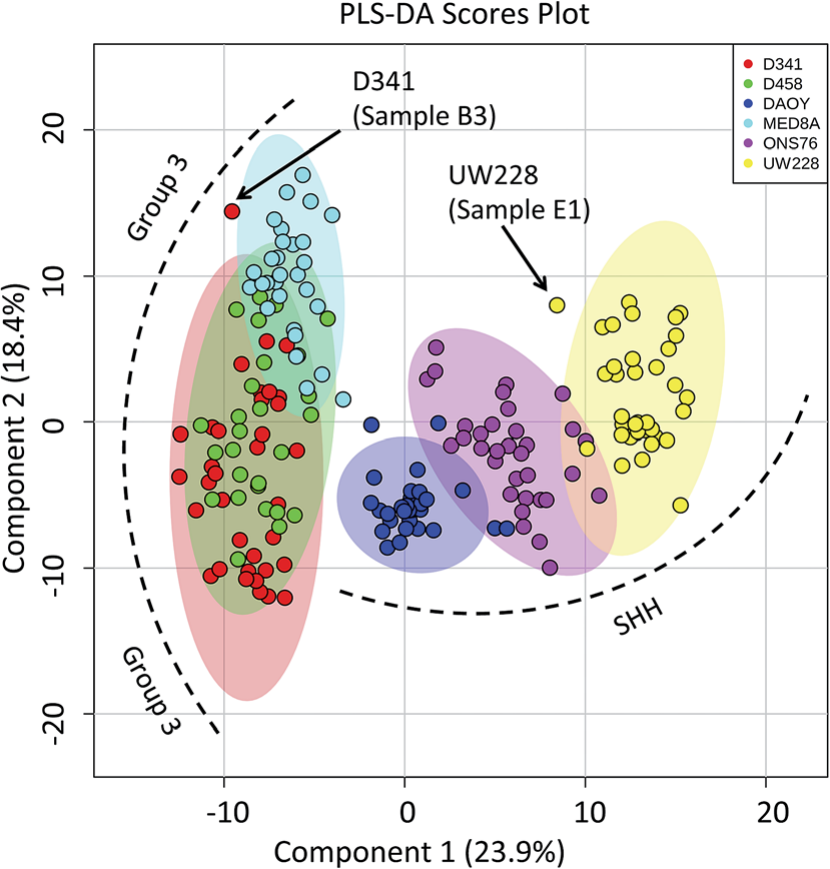
Specificity of PIRL-MS analysis allows statistical discrimination of some MB cell lines based on lipid content. The 194 PIRL-MS spectra of MB xenografts were first grouped based on their respective cell line origins and subjected to a 6-component Partial Least Squares Discriminant Analysis (PLS-DA). Shaded ovals represent the 95% confidence interval. Two outliers are noted that contained weaker than average MS signal. The outlier UW228 sample E1 had only 149 mass peaks identified in its PIRL-MS spectrum, and the weak signal associated with D341, sample B3 (TIC = 6.9 × 10^4^) resulted in only 105 identified peaks. These peak numbers are smaller than those expected for data points that classify well within the model (see ESI†).

Capitalizing on the specificity with which PIRL desorption is able to extract lipids and small molecules from tumours, we examined the possibility of further statistically classifying the MB PIRL-MS dataset based on cell line origin. We thus grouped the 194 PIRL-MS spectra into their 6 respective cell lines, and subjected the dataset thereof to a 6-component PLS-DA assessment. Fig. 3 shows the PLS-DA scores plot for this analysis that ranks each cell line dataset based on multivariate discriminant analysis. The datasets that overlap in occupying the same area in the PLS-DA scores plot are considered statistically indistinguishable. As seen in Fig. 3, with the exception of only two outliers, some of the PIRL-MS data points of individual MB cell lines show distinct statistical grouping. Most pronounced are the DAOY and UW228 cell lines that exhibit a drastically different grouping within the SHH subgroup.

The data points from the ONS76 cell line are shown to separate well from the PIRL-MS data points of the other two SHH cell lines. The results for the Group 3 cell lines were slightly different. Here, the D341 and D458 were essentially identical from the statistical point of view, and the MED8A cell line also shows some degree of lipid profile overlap with the other two Group 3 cell lines examined. The lack of a 1 to 1 correspondence between a genomic profile and its small molecule metabolite or lipid subsets make a direct comparison between PIRL-MS and tumour DNA profiles difficult.^38^ However, with small molecule profiling approaches of this kind becoming accessible in MS studies, new strategies to compare small molecule profiles to genomic information for validation are being developed.^39^ Nevertheless, the separation of cell line data seen here attests to the high specificity of PIRL-MS profiling in tissue identification.

### Caveats, outlook and future directions

**Fig. 4:**
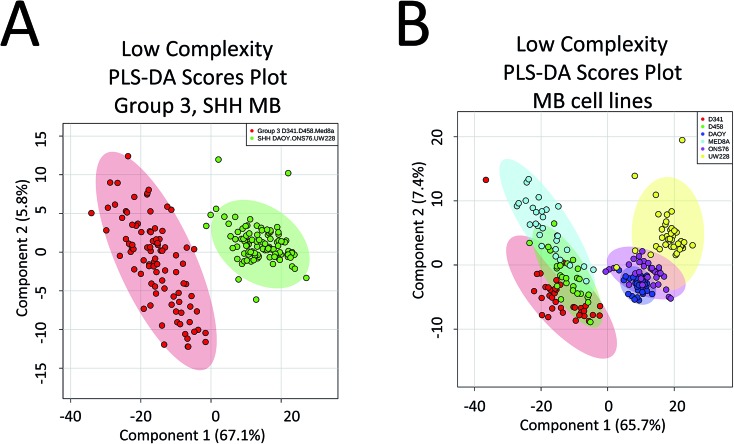
Low complexity Partial Least Squares Discriminant Analysis (PLS-DA) suggests that the discovered biomarker ions are robust determinants of MB subgroup affiliation. Here we performed PLS-DA assessment of the statistical discrimination between Group 3 and SHH subgroups (A) as well as between the 6 MB cell lines of D341, D458, MED8A, DAOY, ONS76 and UW228 (B) using only ∼30 *m*/*z* values listed in Table 1 as biomarker ions for SHH and Group 3 MB. As illustrated here, in both cases, this reduced complexity assessment resulted in approximately the same pattern of statistical separation seen in both Fig. 2 and 3 using the full *m*/*z* range. This observation further suggests that the lower than 50% data utilization in components 1, 2 of the full *m*/*z* range PLS-DA shown in Fig. 2 and 3 is not due to harboring noise. This assessment used a mass tolerance of 25 mDa after post process correction of mass shift using internal mass lock, as described in the method section.

There are a number of technical caveats with our study as described and addressed below. First, due to lack of availability of established WNT and Group 4 cell lines, we only focused on the determination of subgroup affiliation of SHH and Group 3 MB.^40^ Therefore, a comprehensive assessment of the utility of PIRL-MS in all MB subgroup affiliation is lacking. Second, we only used subcutaneous tumours that did not contain healthy brain tissue. An attempt was made to utilize orthotopic xenograft models obtained after intracranial injection of tumour MB cells in the cerebellum of mice. However, the very small size of the resultant tumours made visual targeting of the laser tip to the tumour site nearly impossible in the absence of a suitable imaging and surgical guidance system. In this study, subcutaneous SHH and Group 3 MB samples are shown to possess characteristic PIRL-MS profiles in the absence of potential confounding effects of infiltrating normal brain matter. This study will be expanded into human samples using a local tissue bank where we will compare the PIRL-MS profile of MB tumours to that of the normal posterior fossa tissue. Human samples are also expected to possess more heterogeneity compared to xenograft counterparts. With respect to the confounding effect of sample heterogeneity that is largely lacking in xenograft models (third caveat), in Fig. 4 we show that a low complexity PLS-DA analysis that only utilizes ∼30 *m*/*z* values identified in Table 1 as specific biomarker ions for SHH and Group 3 MB is sufficient to statistically distinguish between cell lines of these two subgroups. This analysis only uses MB specific *m*/*z* values to provide statistical discrimination and not the entire *m*/*z* range of PIRL-MS profiles that could harbor signatures of sample heterogeneity. The separation seen in Fig. 4 suggests that the biomarker ions reported in Table 1 and identified with LC-MS can serve as robust determinants of MB subgroup affiliation. In the absence of significant ion suppression,^41^ the influence of sample heterogeneity on the abundance of MB specific biomarker ions may be small. In case a PIRL-MS data point is obtained through a desorption event from a region that contains non-MB heterogeneity, we expect the reduced complexity assessment described here to be highly sensitive to such a change, providing a red flag for data point exclusion on the basis of drastic mismatch between the expected and the observed reduced complexity PIRL-MS profiles. Such exclusion may be difficult to ascertain using the entire *m*/*z* range due to low sensitivity to change in molecular composition. Our observation here may also open up the use in tumour grading of simpler detection platforms with reduced multiplexing capabilities compared to full size mass spectrometers. Further, it may be possible to classify MB patient tissues based on a xenograft molecular library using the low complexity assessment described in Fig. 4. While this hypothesis must be further validated, it may open up the possibility of creating clinically suitable molecular fingerprint libraries using the more easily accessible, homogenous xenograft models of human cancers.

In conclusion, we have shown that through 5–10 second of sampling with PIRL-MS it is possible to distinguish xenografts of Group 3 MB from the SHH subgroup. Further validation using human samples are required to examine clinical utility. Pursuant to other MS-based methods of rapid intraoperative *ex vivo* tissue characterization, we are currently analyzing the DESI-MS spectra of xenograft MB tumour smears using similar multivariate methods to examine the possibility of ∼1 min subgroup affiliation determination using biopsy smears and non-laser based MS methods.^17,19^

## Author contribution

AZA and HG designed research. MW, JZ, DD, TK, AA performed research and analyzed results. IF, CMKF, MW, SI designed appropriate preclinical models, and performed research. HG, SD, MT, JR contributed to supervisory and provided clinical input. AZA provided supervisory input, and wrote the primary draft of the manuscript. All authors contributed to writing and editing of the manuscript. AZA, HG, SD, JR, MD, AA provided funding for this work.

## References

[cit1] Northcott P. A., Korshunov A., Witt H., Hielscher T., Eberhart C. G., Mack S., Bouffet E., Clifford S. C., Hawkins C. E., French P., Rutka J. T., Pfister S., Taylor M. D. (2011). J. Clin. Oncol..

[cit2] Ramaswamy V., Remke M., Bouffet E., Bailey S., Clifford S. C., Doz F., Kool M., Dufour C., Vassal G., Milde T., Witt O., von Hoff K., Pietsch T., Northcott P. A., Gajjar A., Robinson G. W., Padovani L., Andre N., Massimino M., Pizer B., Packer R., Rutkowski S., Pfister S. M., Taylor M. D., Pomeroy S. L. (2016). Acta Neuropathol..

[cit3] Sabha N., Knobbe C. B., Maganti M., Al Omar S., Bernstein M., Cairns R., Cako B., von Deimling A., Capper D., Mak T. W., Kiehl T. R., Carvalho P., Garrett E., Perry A., Zadeh G., Guha A., Sidney C. (2014). Neuro-Oncology.

[cit4] Gottardo N. G., Hansford J. R., McGlade J. P., Alvaro F., Ashley D. M., Bailey S., Baker D. L., Bourdeaut F., Cho Y. J., Clay M., Clifford S. C., Cohn R. J., Cole C. H., Dallas P. B., Downie P., Doz F., Ellison D. W., Endersby R., Fisher P. G., Hassall T., Heath J. A., Hii H. L., Jones D. T., Junckerstorff R., Kellie S., Kool M., Kotecha R. S., Lichter P., Laughton S. J., Lee S., McCowage G., Northcott P. A., Olson J. M., Packer R. J., Pfister S. M., Pietsch T., Pizer B., Pomeroy S. L., Remke M., Robinson G. W., Rutkowski S., Schoep T., Shelat A. A., Stewart C. F., Sullivan M., Taylor M. D., Wainwright B., Walwyn T., Weiss W. A., Williamson D., Gajjar A. (2014). Acta Neuropathol..

[cit5] Ifa D. R., Eberlin L. S. (2016). Clin. Chem..

[cit6] Takats Z., Strittmatter N., McKenzie J. S. (2017). Adv. Cancer Res..

[cit7] Zhang J., Yu W., Suliburk J., Eberlin L. S. (2016). Clin. Chem..

[cit8] Zhang J. L., Yu W. D., Suliburk J., Eberlin L. S. (2016). Clin. Chem..

[cit9] Eberlin L. S., Norton I., Dill A. L., Golby A. J., Ligon K. L., Santagata S., Cooks R. G., Agar N. Y. (2012). Cancer Res..

[cit10] Fenselau C., Heller D. N., Olthoff J. K., Cotter R. J., Kishimoto Y., Uy O. M. (1989). Biomed. Environ. Mass Spectrom..

[cit11] Dill A. L., Ifa D. R., Manicke N. E., Ouyang Z., Cooks R. G. (2009). J. Chromatogr. B: Anal. Technol. Biomed. Life Sci..

[cit12] Eberlin L. S., Dill A. L., Costa A. B., Ifa D. R., Cheng L., Masterson T., Koch M., Ratliff T. L., Cooks R. G. (2010). Anal. Chem..

[cit13] Dill A. L., Eberlin L. S., Zheng C., Costa A. B., Ifa D. R., Cheng L., Masterson T. A., Koch M. O., Vitek O., Cooks R. G. (2010). Anal. Bioanal. Chem..

[cit14] Dill A. L., Eberlin L. S., Costa A. B., Zheng C., Ifa D. R., Cheng L., Masterson T. A., Koch M. O., Vitek O., Cooks R. G. (2011). Chemistry.

[cit15] Gerbig S., Golf O., Balog J., Denes J., Baranyai Z., Zarand A., Raso E., Timar J., Takats Z. (2012). Anal. Bioanal. Chem..

[cit16] Eberlin L. S., Norton I., Orringer D., Dunn I. F., Liu X., Ide J. L., Jarmusch A. K., Ligon K. L., Jolesz F. A., Golby A. J., Santagata S., Agar N. Y., Cooks R. G. (2013). Proc. Natl. Acad. Sci. U. S. A..

[cit17] Jarmusch A. K., Pirro V., Baird Z., Hattab E. M., Cohen-Gadol A. A., Cooks R. G. (2016). Proc. Natl. Acad. Sci. U. S. A..

[cit18] Wiseman J. M., Ifa D. R., Song Q., Cooks R. G. (2006). Angew. Chem., Int. Ed..

[cit19] Woolman M., Tata A., Bluemke E., Dara D., Ginsberg H. J., Zarrine-Afsar A. (2017). J. Am. Soc. Mass Spectrom..

[cit20] Tata A., Woolman M., Ventura M., Bernards N., Ganguly M., Gribble A., Shrestha B., Bluemke E., Ginsberg H. J., Vitkin A., Zheng J., Zarrine-Afsar A. (2016). Sci. Rep..

[cit21] Balog J., Sasi-Szabo L., Kinross J., Lewis M. R., Muirhead L. J., Veselkov K., Mirnezami R., Dezso B., Damjanovich L., Darzi A., Nicholson J. K., Takats Z. (2013). Sci. Transl. Med..

[cit22] Balog J., Szaniszlo T., Schaefer K. C., Denes J., Lopata A., Godorhazy L., Szalay D., Balogh L., Sasi-Szabo L., Toth M., Takats Z. (2010). Anal. Chem..

[cit23] Sachfer K. C., Szaniszlo T., Gunther S., Balog J., Denes J., Keseru M., Dezso B., Toth M., Spengler B., Takats Z. (2011). Anal. Chem..

[cit24] Schafer K. C., Balog J., Szaniszlo T., Szalay D., Mezey G., Denes J., Bognar L., Oertel M., Takats Z. (2011). Anal. Chem..

[cit25] Kwiatkowski M., Wurlitzer M., Krutilin A., Kiani P., Nimer R., Omidi M., Mannaa A., Bussmann T., Bartkowiak K., Kruber S., Uschold S., Steffen P., Lubberstedt J., Kupker N., Petersen H., Knecht R., Hansen N. O., Zarrine-Afsar A., Robertson W. D., Miller R. J., Schluter H. (2016). J. Proteomics.

[cit26] Amini-Nik S., Kraemer D., Cowan M. L., Gunaratne K., Nadesan P., Alman B. A., Miller R. J. (2010). PLoS One.

[cit27] Zou J., Talbot F., Tata A., Ermini L., Franjic K., Ventura M., Zheng J., Ginsberg H., Post M., Ifa D. R., Jaffray D., Miller R. J., Zarrine-Afsar A. (2015). Anal. Chem..

[cit28] Franjic K., Miller D. (2010). Phys. Chem. Chem. Phys..

[cit29] Cowan M. L., Bruner B. D., Huse N., Dwyer J. R., Chugh B., Nibbering E. T., Elsaesser T., Miller R. J. (2005). Nature.

[cit30] Franjic K., Cowan M. L., Kraemer D., Miller R. J. (2009). Opt. Express.

[cit31] Woolman M., Gribble A., Bluemke E., Zou J., Ventura M., Bernards N., Wu M., Ginsberg H. J., Das S., Vitkin A., Zarrine-Afsar A. (2017). Sci. Rep..

[cit32] Ermini L., Morganti E., Post A., Yeganeh B., Caniggia I., Leadley M., Faria C. C., Rutka J. T., Post M. (2017). PLoS One.

[cit33] Staal J. A., Lau L. S., Zhang H., Ingram W. J., Hallahan A. R., Northcott P. A., Pfister S. M., Wechsler-Reya R. J., Rusert J. M., Taylor M. D., Cho Y. J., Packer R. J., Brown K. J., Rood B. R. (2015). OncoTargets Ther..

[cit34] Fahy E., Sud M., Cotter D., Subramaniam S. (2007). Nucleic Acids Res..

[cit35] Worley B., Powers R. (2013). Curr. Metabolomics.

[cit36] Tang X., Lin C. C., Spasojevic I., Iversen E. S., Chi J. T., Marks J. R. (2014). Breast Cancer Res..

[cit37] Fahy E., Subramaniam S., Murphy R. C., Nishijima M., Raetz C. R., Shimizu T., Spener F., van Meer G., Wakelam M. J., Dennis E. A. (2009). J. Lipid Res..

[cit38] Griffin J. L., Shockcor J. P. (2004). Nat. Rev. Cancer.

[cit39] Desai D. K., Schunck H., Loser J. W., Laroche J. (2013). Bioinformatics.

[cit40] Ivanov D. P., Coyle B., Walker D. A., Grabowska A. M. (2016). J. Biotechnol..

[cit41] Furey A., Moriarty M., Bane V., Kinsella B., Lehane M. (2013). Talanta.

