# Supplementary material for: Rapid determination of medulloblastoma subgroup affiliation with mass spectrometry using a handheld picosecond infrared laser desorption probe
Source: Chem Sci. 2017 Jul 21;8(9):6508–19. doi: 10.1039/c7sc01974b (PMC5628578; doi:10.1039/c7sc01974b)
Supplement: Supplementary file 1 [file SC-008-C7SC01974B-s001.pdf]

Electronic Supplementary Material (ESI) for Chemical Science.
This journal is © The Royal Society of Chemistry 2017

Electronic Supplementary Information (ESI)

Rapid Determination of Medulloblastoma Subgroup Affiliation With Mass Spectrometry Using a Hand-held Picosecond Infrared Laser Desorption Probe

Michael Woolman[a,b], Isabelle Ferry[c,h,i,*], Claudia M. Kuzan-Fischer[c,hi,*], Megan Wu[c,h,i*], Jing Zou[a], Taira Kiyota[d], Semra Isik[c], Delaram Dara[a] Ahmed Aman[d], Sunit Das[c,e,f], Michael D. Taylor[c,e,h,i], James T. Rutka[c,e,h] , Howard J. Ginsberg[a,e,f,g], and Arash Zarrine-Afsar [a,b,e,f ,**]

[a] Techna Institute for the Advancement of Technology for Health, University Health Network, 100 College Street, Toronto, ON, M5G 1P5, Canada
[b] Department of Medical Biophysics, University of Toronto, 101 College Street, Toronto, ON, M5G 1L7, Canada
[c] Peter Gilgan Centre for Research and Learning, Hospital for Sick Children, 686 Bay Street, Toronto, ON, M5G 0A4 , Canada
[d] Drug Discovery Program, Ontario Institute for Cancer Research, 661 University Avenue, Toronto, ON M5G 0A3, Canada
[e] Department of Surgery, University of Toronto, 149 College Street, Toronto, ON, M5T 1P5, Canada
[f] Keenan Research Center for Biomedical Science & the Li Ka Shing Knowledge Institute, St. Michael's Hospital, 30 Bond Street, Toronto, ON, M5B 1W8, Canada
[g] Institute of Biomaterials and Biomedical Engineering, University of Toronto, 164 College Street, Toronto, ON, M5S 3G9, Canada
[h] Arthur and Sonia Labatt Brain Tumor Research Centre, The Hospital for Sick Children, Toronto, ON, M5G 1X8, Canada
[i] Developmental & Stem Cell Biology Program, The Hospital for Sick Children, 686 Bay Street, Toronto, ON, M5G 0A4 , Canada

* These authors contributed equally to this work
**Corresponding author: arash.zarrine.afsar@utoronto.ca

Table of Contents

Figure S1………..Page 3
Figure S2………Pages 4-7
Figure S3………..Page 8
Figure S4……..Pages 9-17
Figure S5………Page 19
Analytical performance and the duty cycle…………………………………………………………………..Page 20
References……Page 22

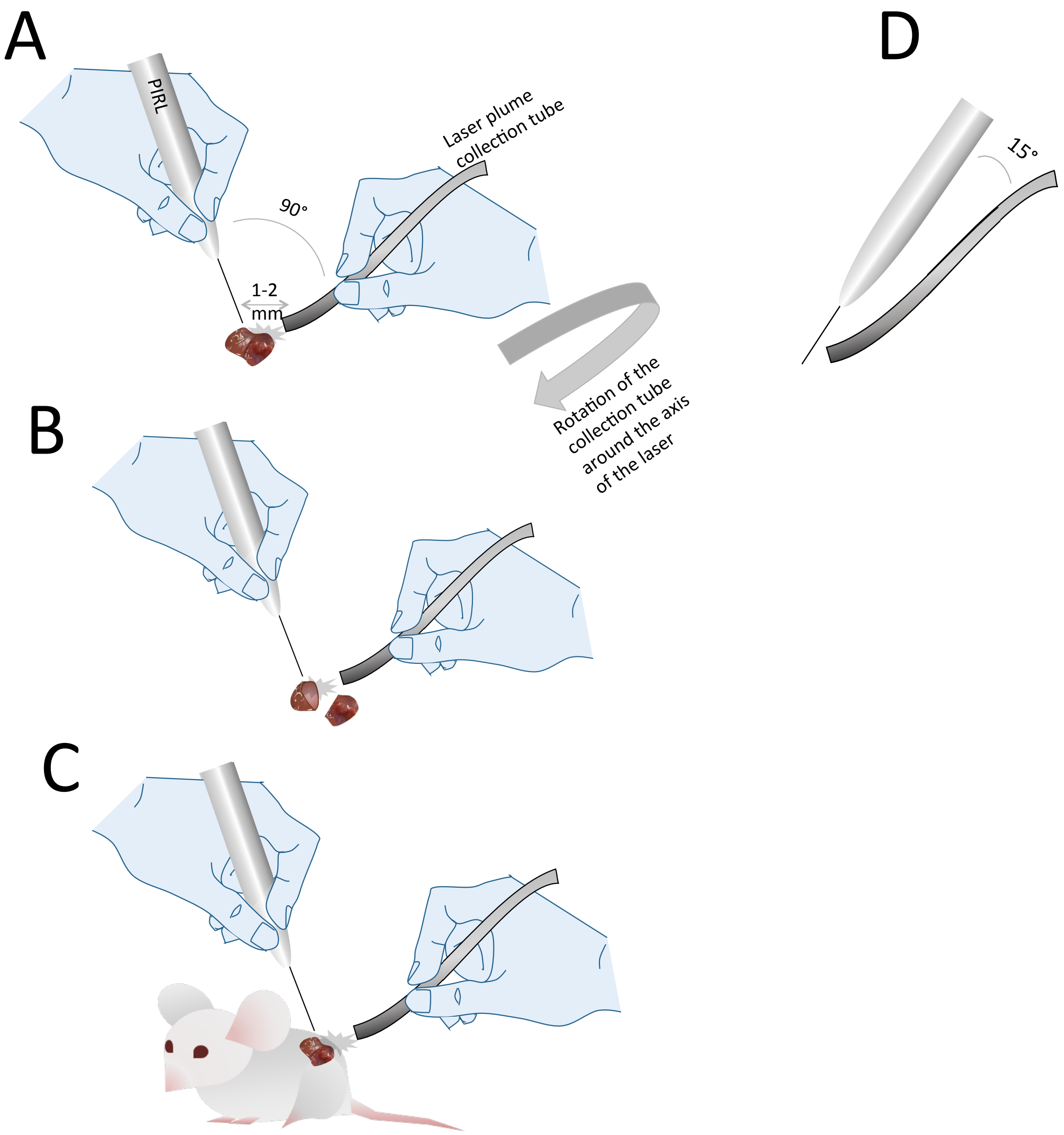

Figure S1. The schematics of the PIRL MS experimental setup for the determination of MB subgroup affiliation. Murine xenograft tumours were surgically exposed, resected and subjected with PIRL MS sampling as *ex vivo* tissue as indicated in A,B for analysis of tumour surface (A) and tumour cores (B). Note that *in situ* sampling as shown in C is also possible with our current platform but was not pursued for the subcutaneous tumours analyzed in this study. The angle between laser tip and collection tube was 90 degrees. This cartoon is partly adapted from our previous publication, replicated here for the clarity of discussion[1]. Fixed geometry shown in (D) was also attempted but was found not to be optimal. The rotation of the collection tube around the axis of the laser tip to optimize the signal is shown in (A).

Figure S2. Statistical robustness of MB subclass prediction with PIRL MS through a 5% leave out and remodel test. Below, we show 21 runs of 3-component Partial Least Squares Discriminant Analysis (PLS-DA) where 10 PIRL MS data points were iteratively taken out (oversampled dataset of 210 points), and ranked as pseudo-unknowns for correct grouping with the expected MB subgroup data from a model constructed from the remainder 95% of the PIRL MS data points. Each run is labelled with a run number accordingly. A total of 12 outlier data points were identified indicating a 94% correct prediction rate. No misclassification of data points was seen, and none of the 21 models exhibited a breakdown with overlaps in 95% confidence interval. Shaded ovals in each panel represent the 95% confidence interval for each data group. Outliers are clearly indicated in each run.

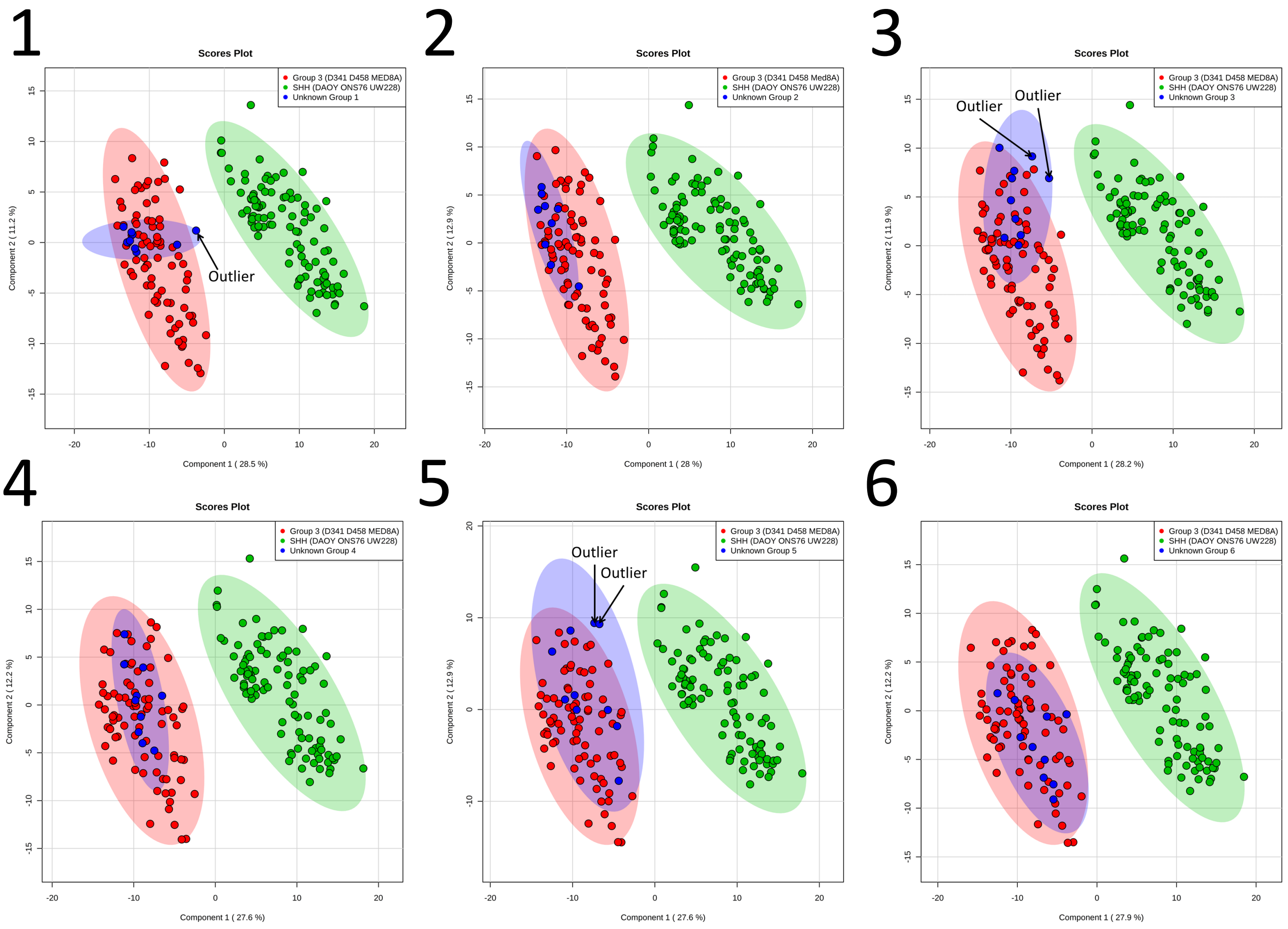
1
Scores Plot
Group 3 (D341 D458 MED8A)
SHH (DAOY ONS76 UW228)
Unknown Group 1
Outlier
Component 1 (28.5 %)
Component 2 (11.2 %)
2
Scores Plot
Group 3 (D341 D458 Med8A)
SHH (DAOY ONS76 UW228)
Unknown Group 2
Component 1 (28 %)
Component 2 (12.9 %)
3
Scores Plot
Group 3 (D341 D458 Med8A)
SHH (DAOY ONS76 UW228)
Unknown Group 3
Outlier
Outlier
Component 1 (28.2 %)
Component 2 (11.9 %)
4
Scores Plot
Group 3 (D341 D458 MED8A)
SHH (DAOY ONS76 UW228)
Unknown Group 4
Component 1 (27.6 %)
Component 2 (12.2 %)
5
Scores Plot
Group 3 (D341 D458 MED8A)
SHH (DAOY ONS76 UW228)
Unknown Group 5
Outlier
Outlier
Component 1 (27.6 %)
Component 2 (12.9 %)
6
Scores Plot
Group 3 (D341 D458 MED8A)
SHH (DAOY ONS76 UW228)
Unknown Group 6
Component 1 (27.9 %)
Component 2 (12.2 %)

7

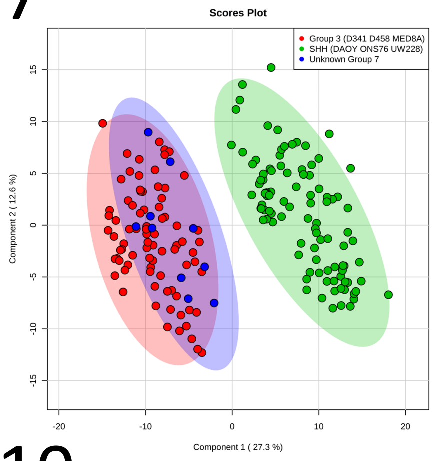
Scores Plot
Group 3 (D341 D458 MED8A)
SHH (DAOY ONS76 UW228)
Unknown Group 7
Component 1 (27.3 %)
Component 2 (12.6 %)

8

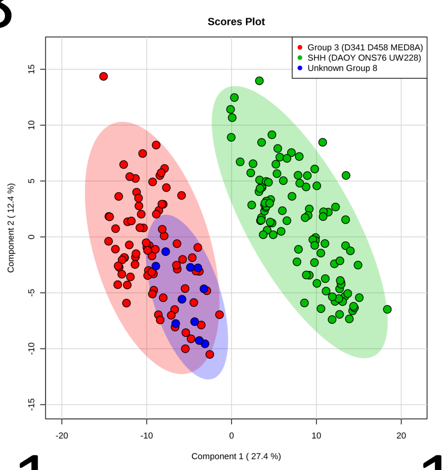
Scores Plot
Group 3 (D341 D458 MED8A)
SHH (DAOY ONS76 UW228)
Unknown Group 8
Component 1 (27.4 %)
Component 2 (12.4 %)

9

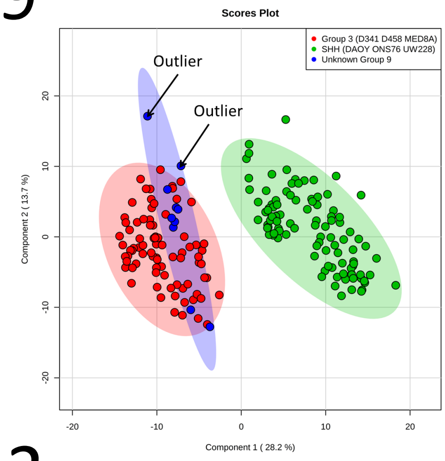
Scores Plot
Outlier
Outlier
Group 3 (D341 D458 MED8A)
SHH (DAOY ONS76 UW228)
Unknown Group 9
Component 1 (28.2 %)
Component 2 (13.7 %)

10

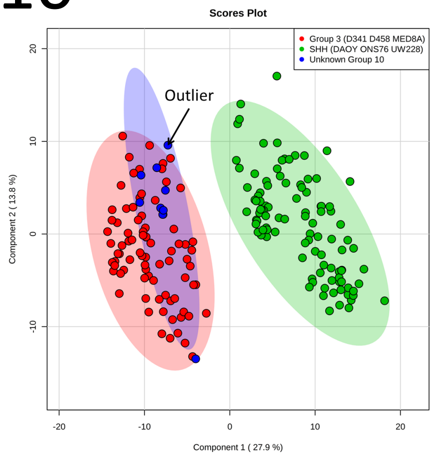
Scores Plot
Outlier
Group 3 (D341 D458 MED8A)
SHH (DAOY ONS76 UW228)
Unknown Group 10
Component 1 (27.9 %)
Component 2 (13.8 %)

11

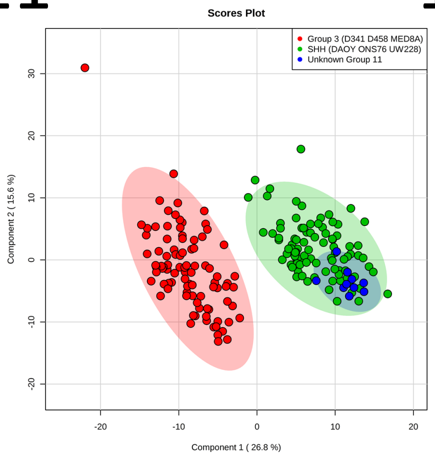
Scores Plot
Group 3 (D341 D458 MED8A)
SHH (DAOY ONS76 UW228)
Unknown Group 11
Component 1 (26.8 %)
Component 2 (15.6 %)

12

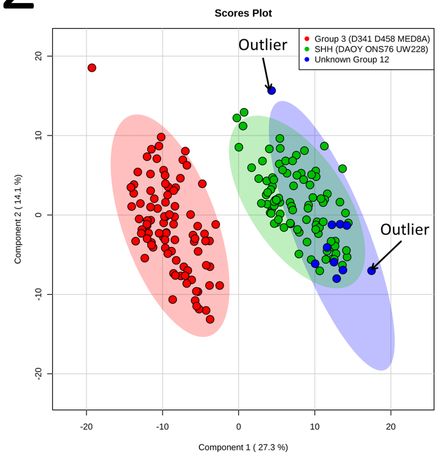
Scores Plot
Outlier
Outlier
Group 3 (D341 D458 MED8A)
SHH (DAOY ONS76 UW228)
Unknown Group 12
Component 1 (27.3 %)
Component 2 (14.1 %)

13

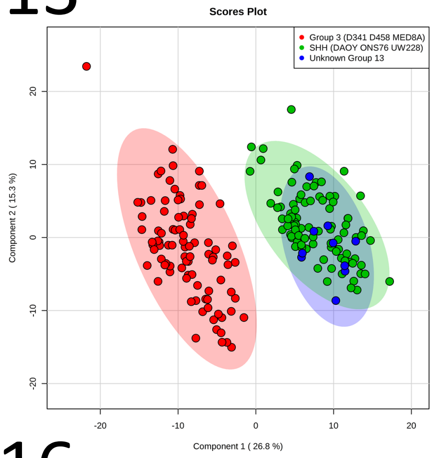

14

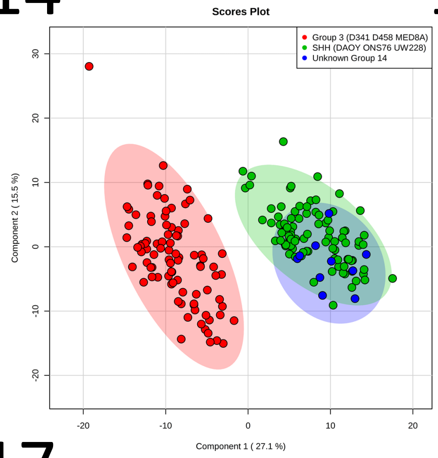

15

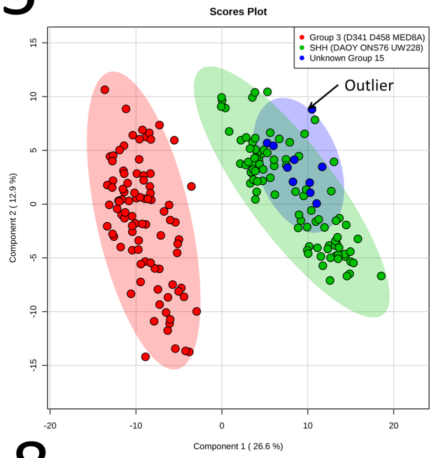

16

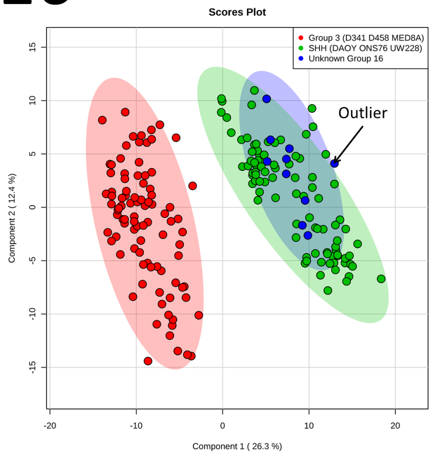

17

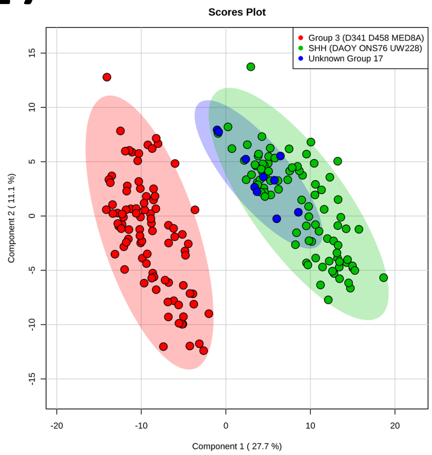

18

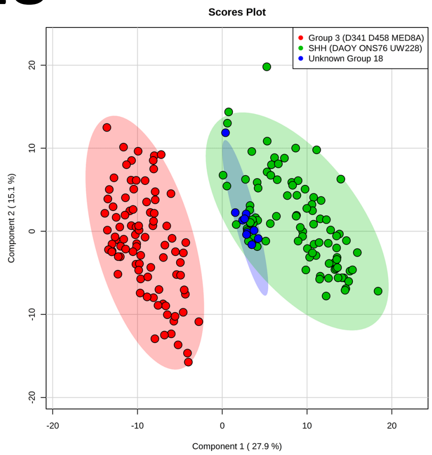

19

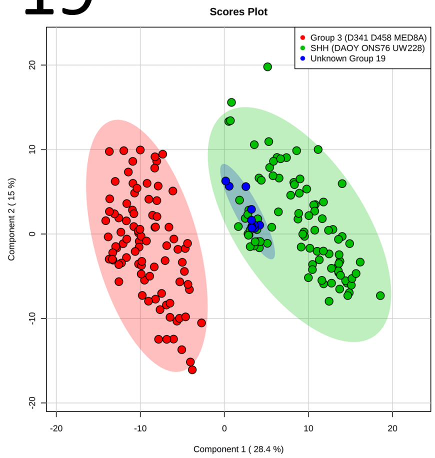

20

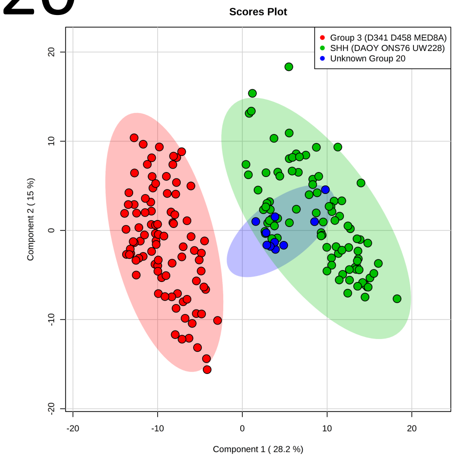

21

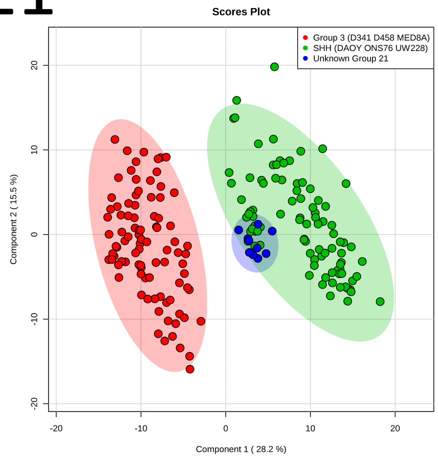

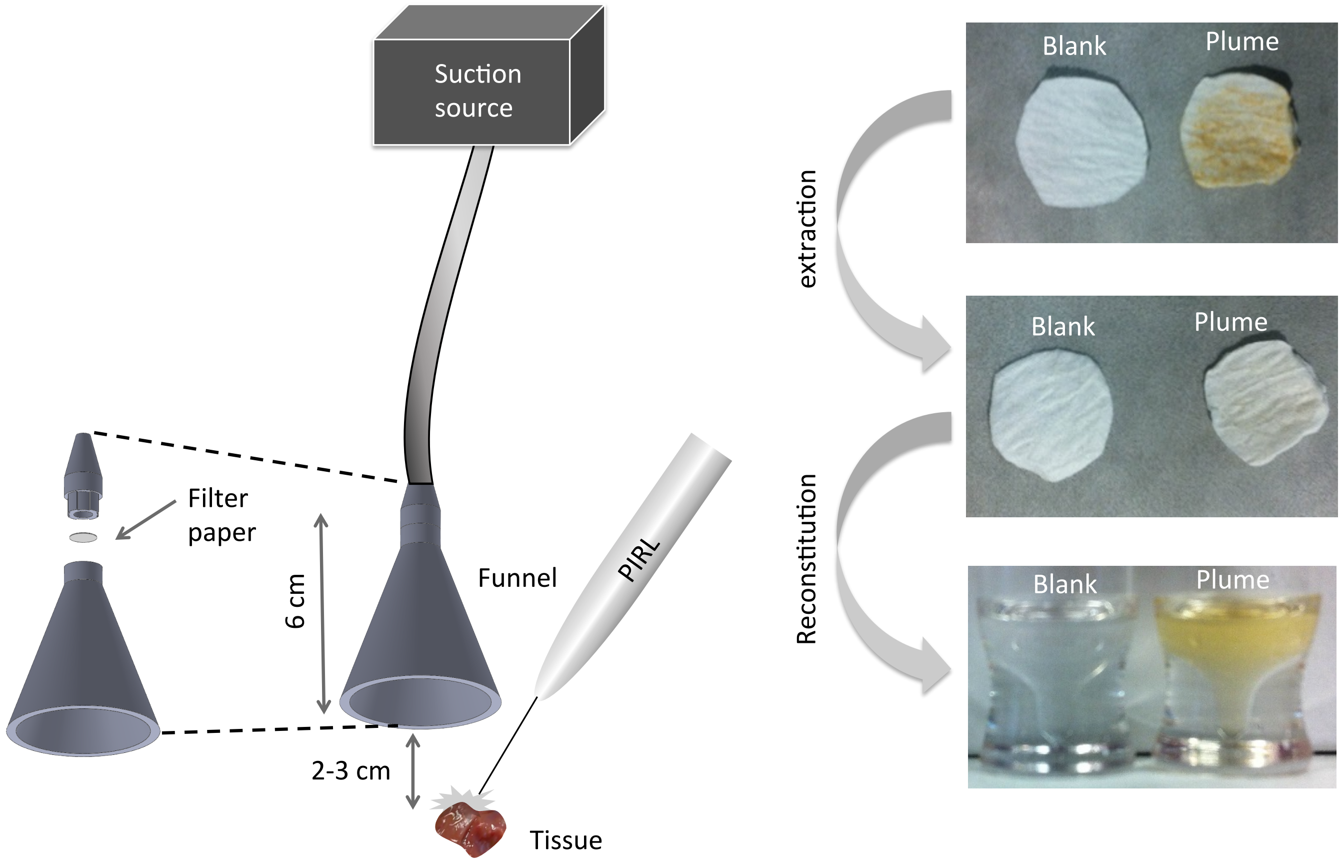

Figure S3. The schematics of the PIRL desorption plume capture system and extraction of lipid molecules. Here, a cellulose filter paper from Whatman was placed in the vacuum collection line interfaced with a funnel. Sampling continued under suction using a Laedral Suction Unit (LSU) for ~ 5 min until the filter paper was visibly stained with the collected plume material. Ablative tissue chunks are highly unlikely to travel all the way to the filter paper. However, a verification of this using high resolution electron microscopy was not performed to confirm capture of gas phase plume as opposed to tissue chunks. For reasons of simplicity, the cartoon of tissue piece/laser is adapted from our previous publication[1]. Panels to the right show the filter paper after collection of plume is visibly stained, and after the removal of plume material with chloroform extraction retains a background colour very similar to the blank filter paper not come in contact with laser desorption plume material. After reconstitution as described in the text, significant amount of the plume material is recovered from the filter paper.

Figure S4. The PLS-DA box plots indicating changes in the abundance of MB classifying *m/z* values between Group 3 and the SHH subtype as indicated by PIRL MS spectra of MED8A and DAOY tumours. Below, box plots from assessment made and presented in Fig. 2 are reported. Both absolute and relative (normalized) ion abundance values are shown. Standard output plots from MetaboAnalyst[2, 3] are reported. The normalized values (to sum of all intensities) are used for our analysis, and are reported in the standard format of box & whisker plot. Here, the bottom and the top of each box indicate the 25th and 75th percentile (also known as the lower and upper quartiles, Q1 and Q3). The line in the middle is the 50th percentile (median, or Q2). The upper whisker indicates the smaller of the maximum value and Q3 + 1.5 IQR (Interquantile Range). The lower whisker is presented at the larger of the smallest variable value and the Q1–1.5 IQR, as above. For each *m/z* value shown in Table 1, those that show no overlap between boxes are marked with "✓" indicating significant change in abundance between the two MB subgroups. Those *m/z* values marked with "✗" on the other hand did not show a significant change in abundance between the two populations and as such cannot be considered a reliable biomarker ion for MB classification. Both "original concentration" and "normalized concentration" from MetaboAnalyst [3, 4]portal are shown that indicate absolute ion abundance and relative ion abundance (normalized to the sum of all ion intensities (total ion count or TIC)) are shown.

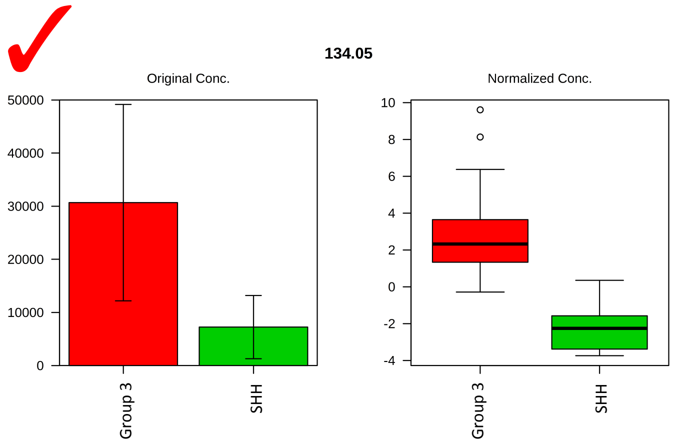
134.05
Original Conc.
Normalized Conc.
Group 3
SHH

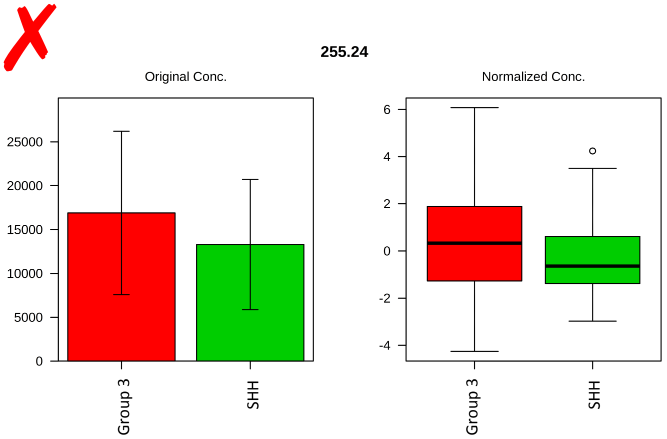
255.24
Original Conc.
Normalized Conc.
Group 3
SHH

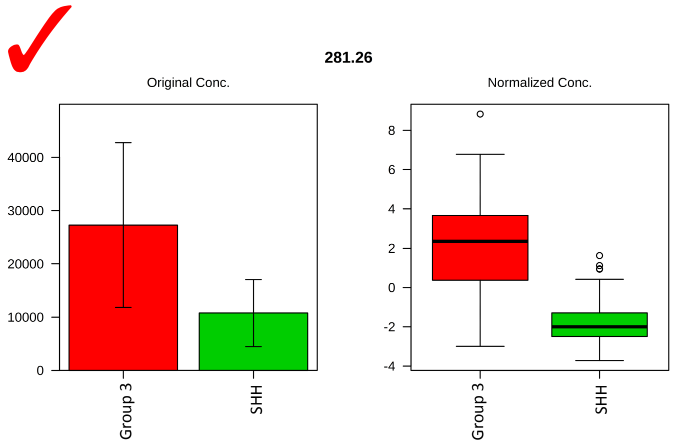
281.26
Original Conc.
Normalized Conc.
Group 3
SHH

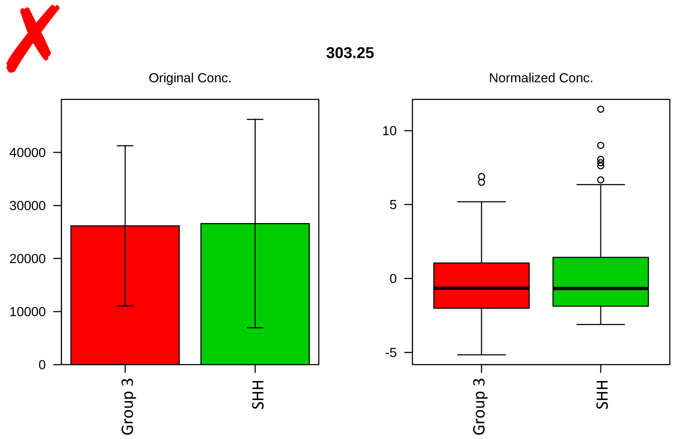
303.25
Original Conc.
Normalized Conc.
Group 3
SHH

✓

305.27

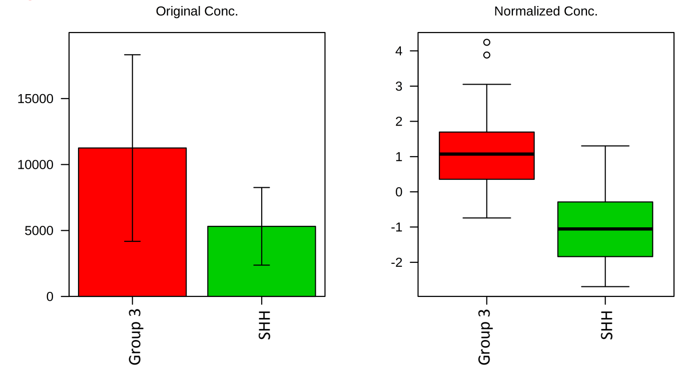

✗

327.25

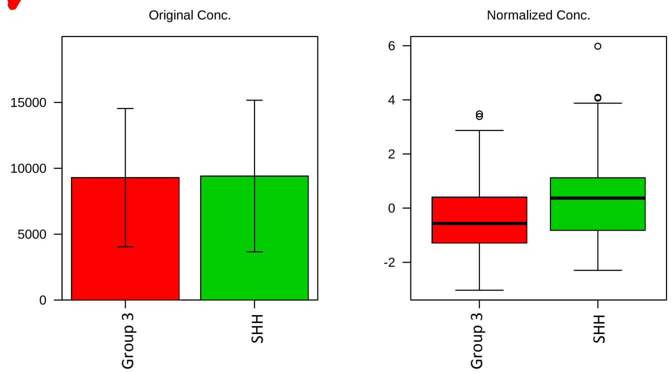

✓

329.26

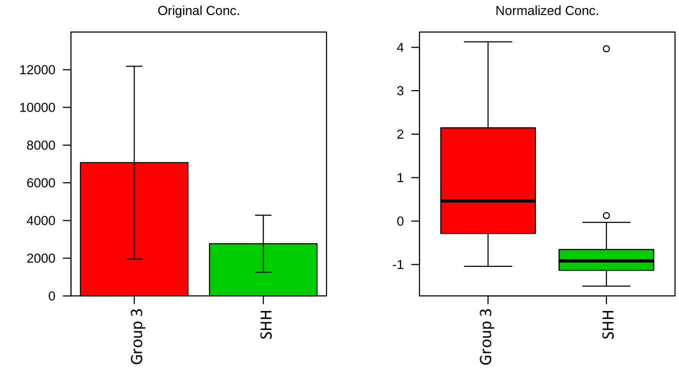

✗

391.24

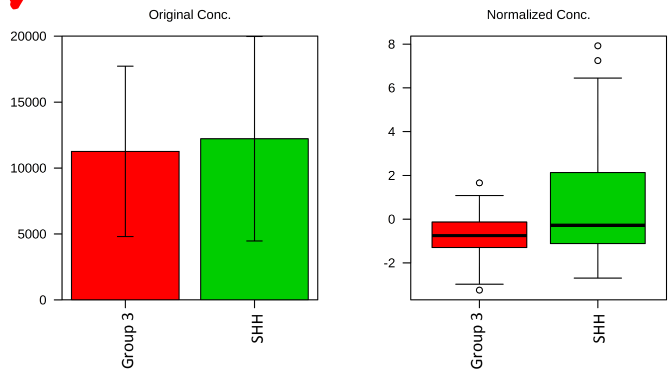

✓ 417.26

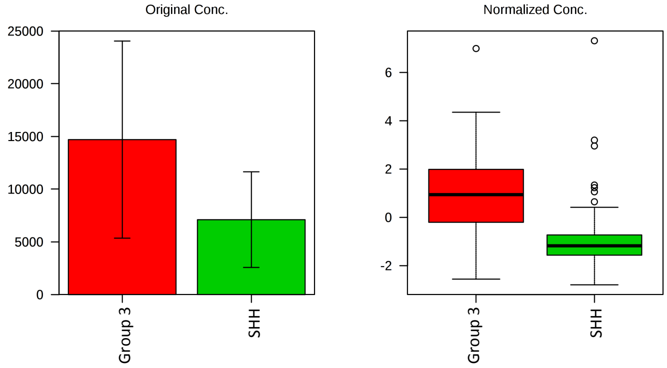

✓ 572.51

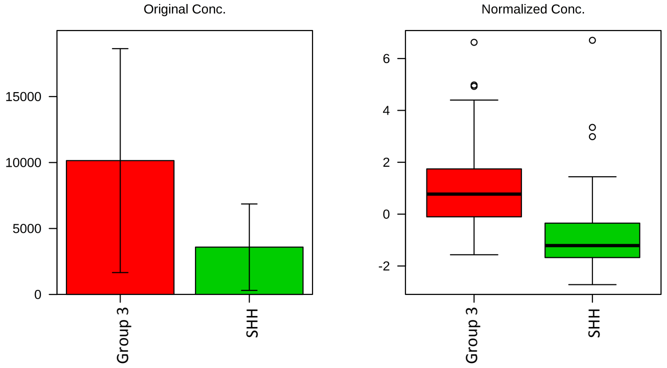

✗ 629.52

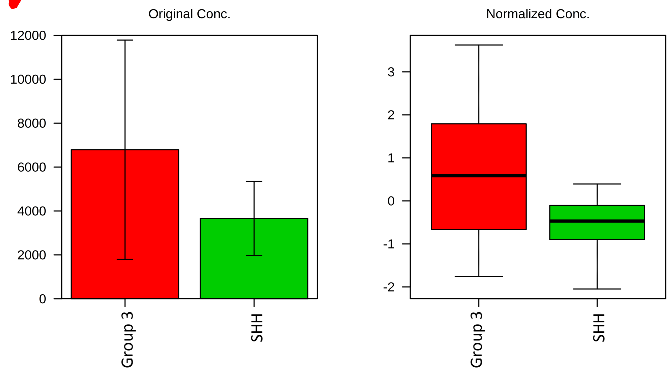

✓ 659.5

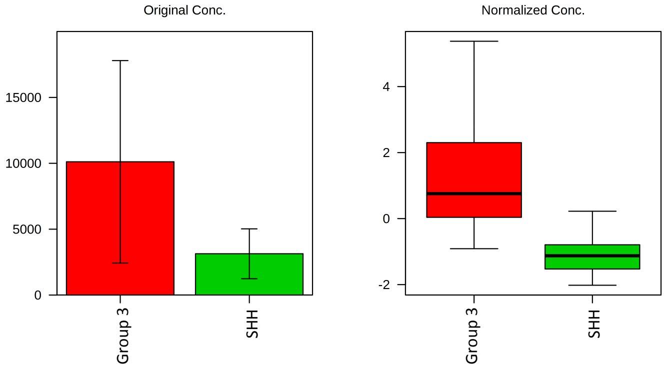

663.5

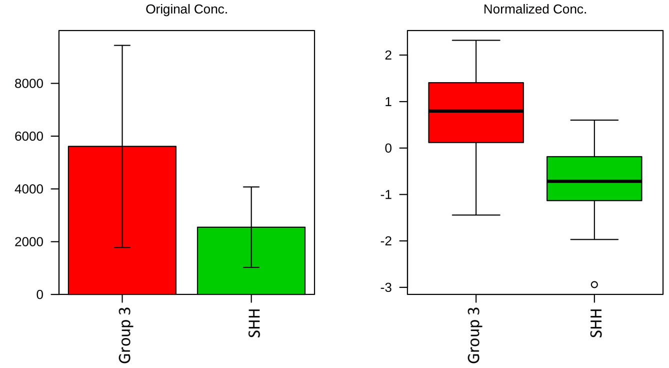

687.52

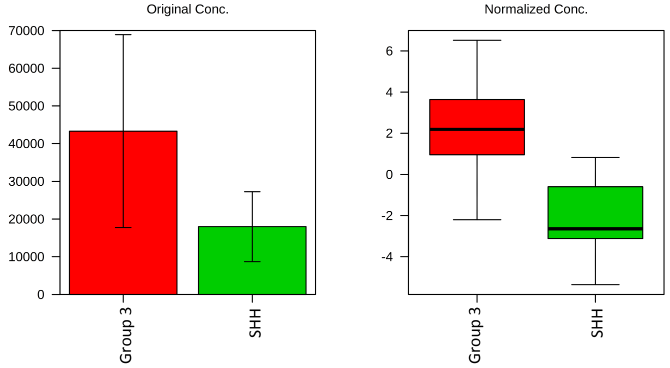

691.52

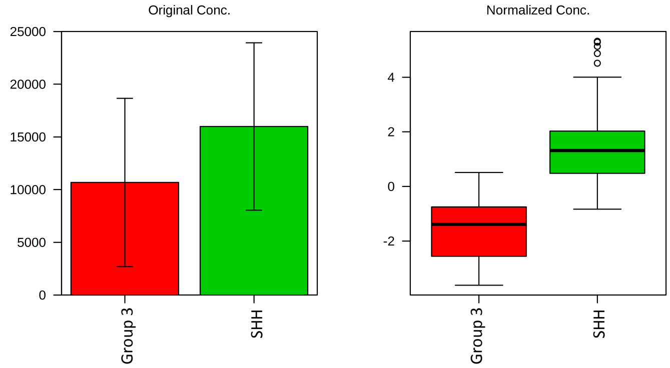

709.51

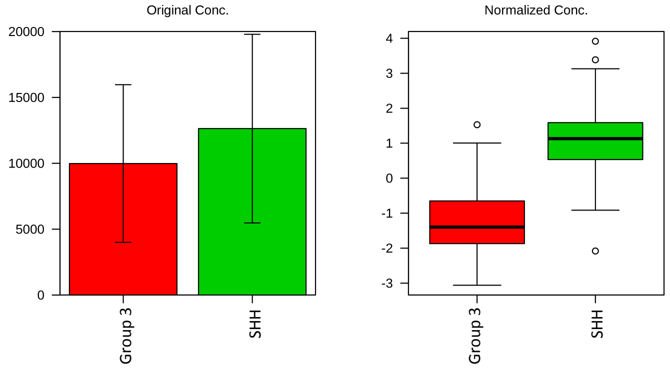

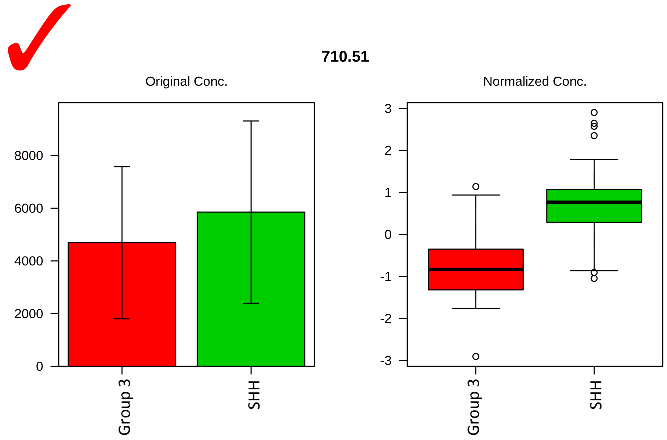
710.51
Original Conc.
Normalized Conc.
Group 3
SHH

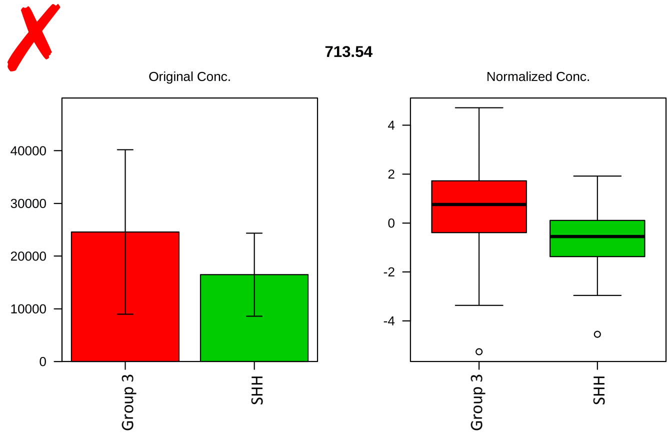
713.54
Original Conc.
Normalized Conc.
Group 3
SHH

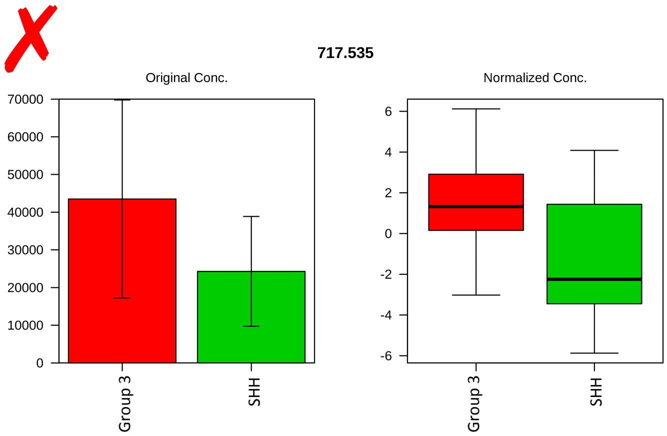
717.535
Original Conc.
Normalized Conc.
Group 3
SHH

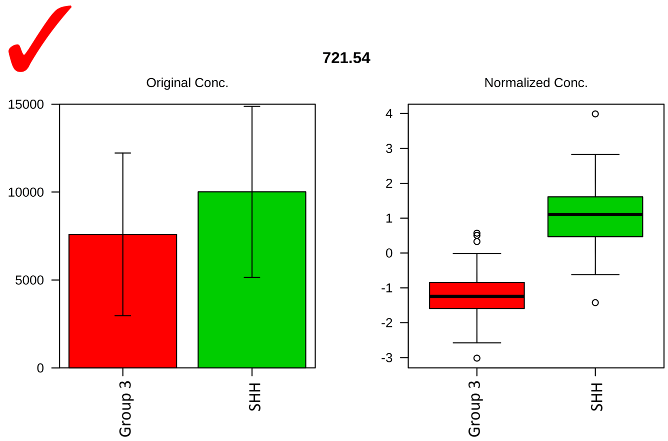
721.54
Original Conc.
Normalized Conc.
Group 3
SHH

723.53

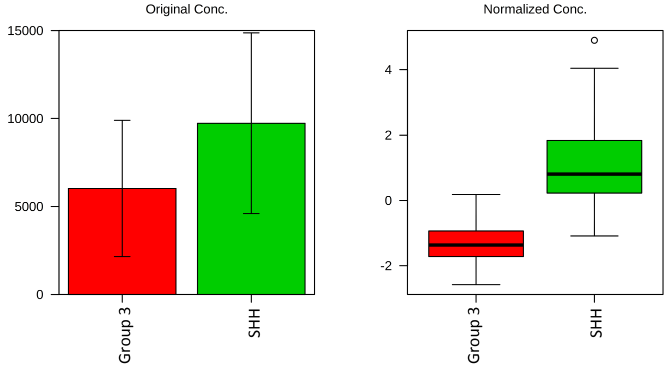

733.51

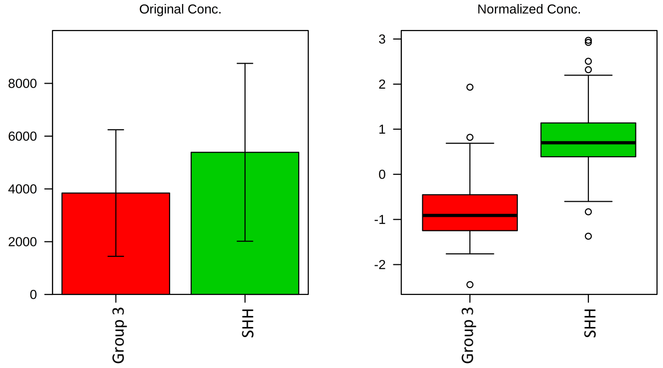

737.54

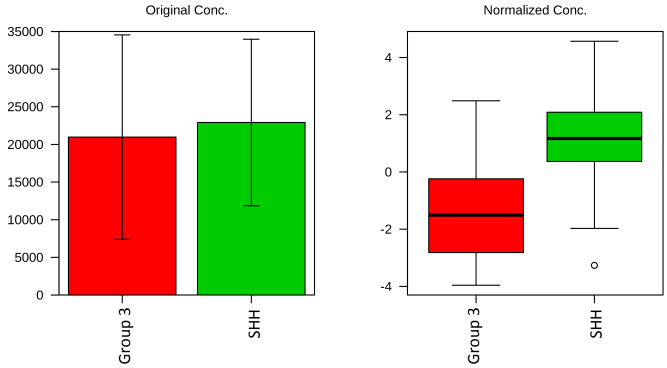

739.53

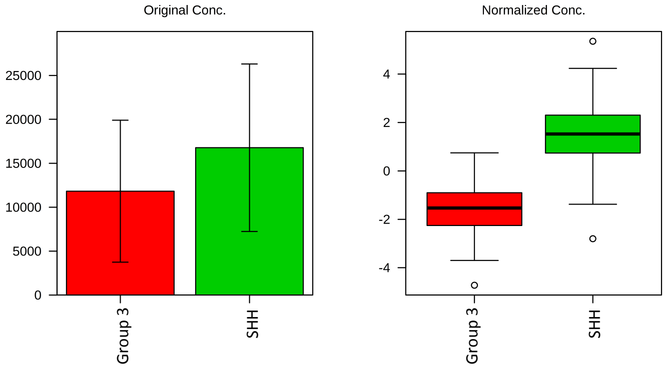

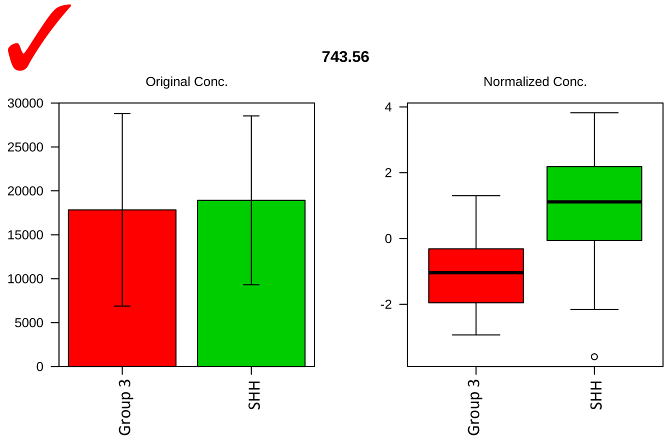
743.56
Original Conc.
Normalized Conc.
30000
25000
20000
15000
10000
5000
0
4
2
0
-2
Group 3
SHH
Group 3
SHH

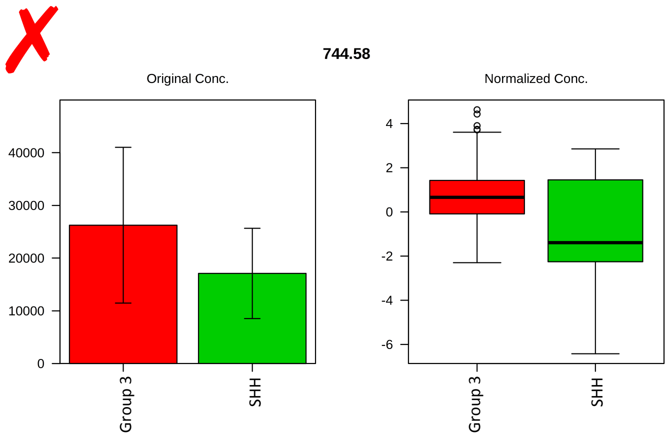
744.58
Original Conc.
Normalized Conc.
40000
30000
20000
10000
0
4
2
0
-2
-4
-6
Group 3
SHH
Group 3
SHH

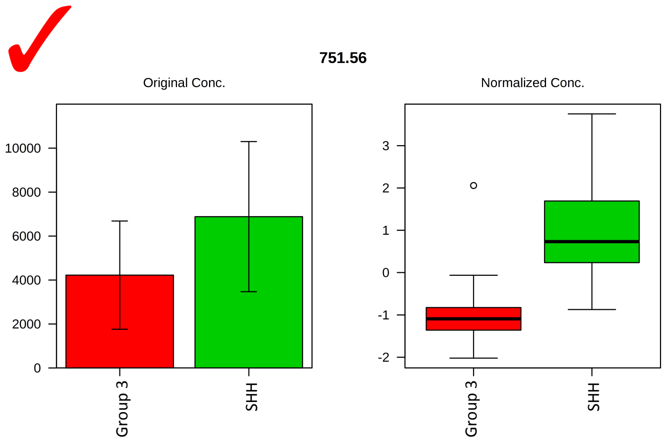
751.56
Original Conc.
Normalized Conc.
10000
8000
6000
4000
2000
0
3
2
1
0
-1
-2
Group 3
SHH
Group 3
SHH

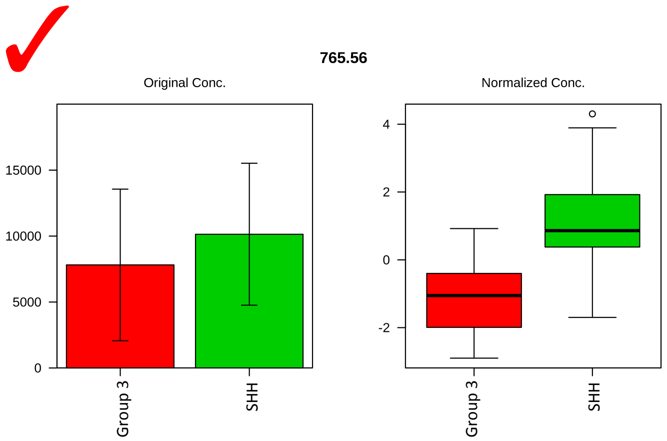
765.56
Original Conc.
Normalized Conc.
15000
10000
5000
0
4
2
0
-2
Group 3
SHH
Group 3
SHH

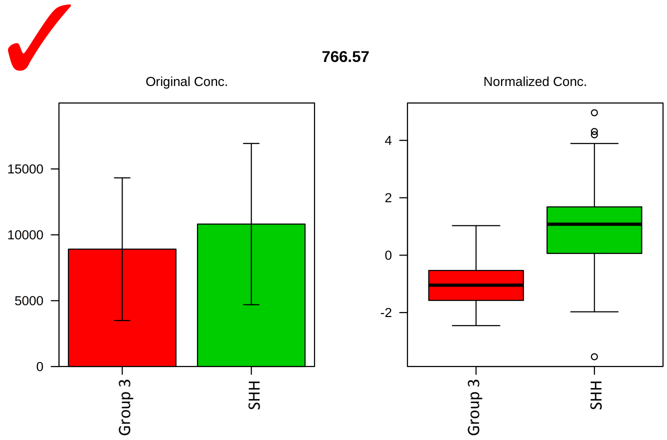
766.57
Original Conc.
Normalized Conc.
Group 3
SHH

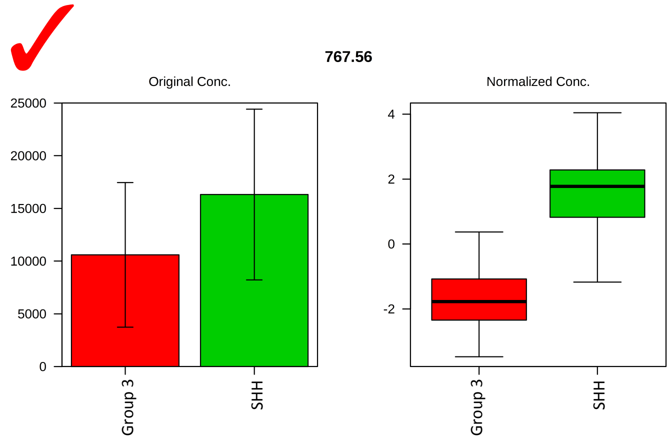
767.56
Original Conc.
Normalized Conc.
Group 3
SHH

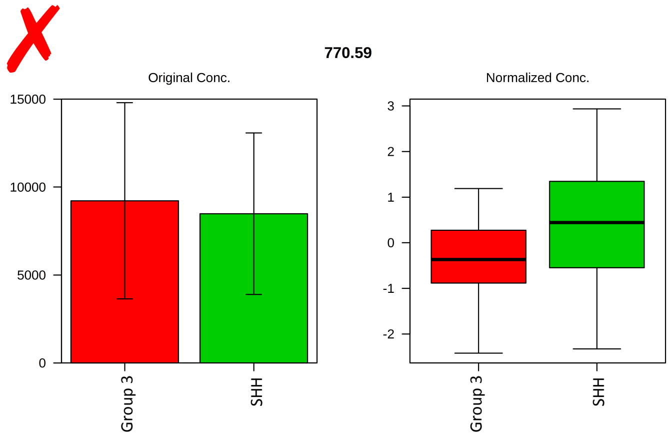
770.59
Original Conc.
Normalized Conc.
Group 3
SHH

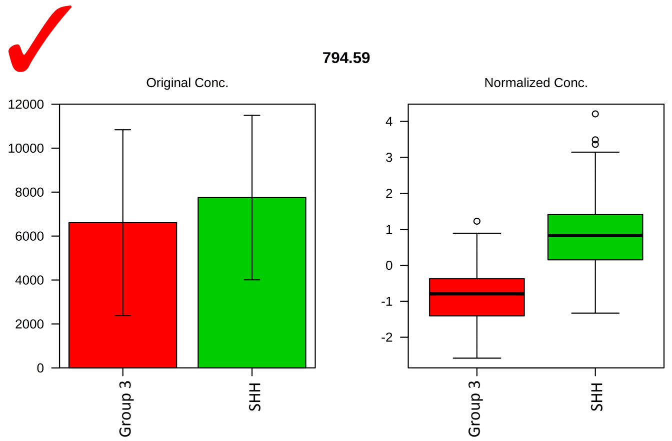
794.59
Original Conc.
Normalized Conc.
Group 3
SHH

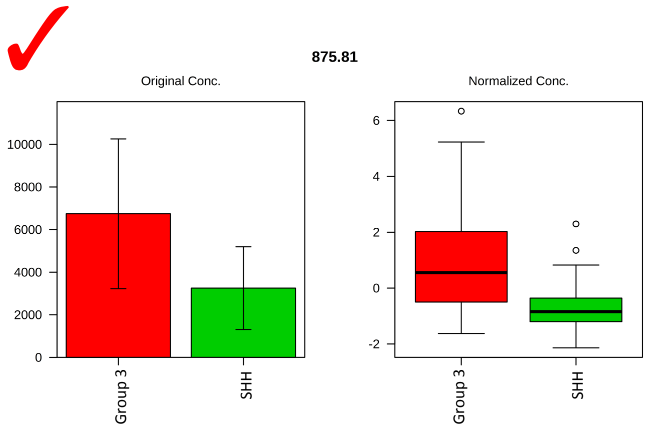
875.81
Original Conc.
Normalized Conc.
Group 3
SHH

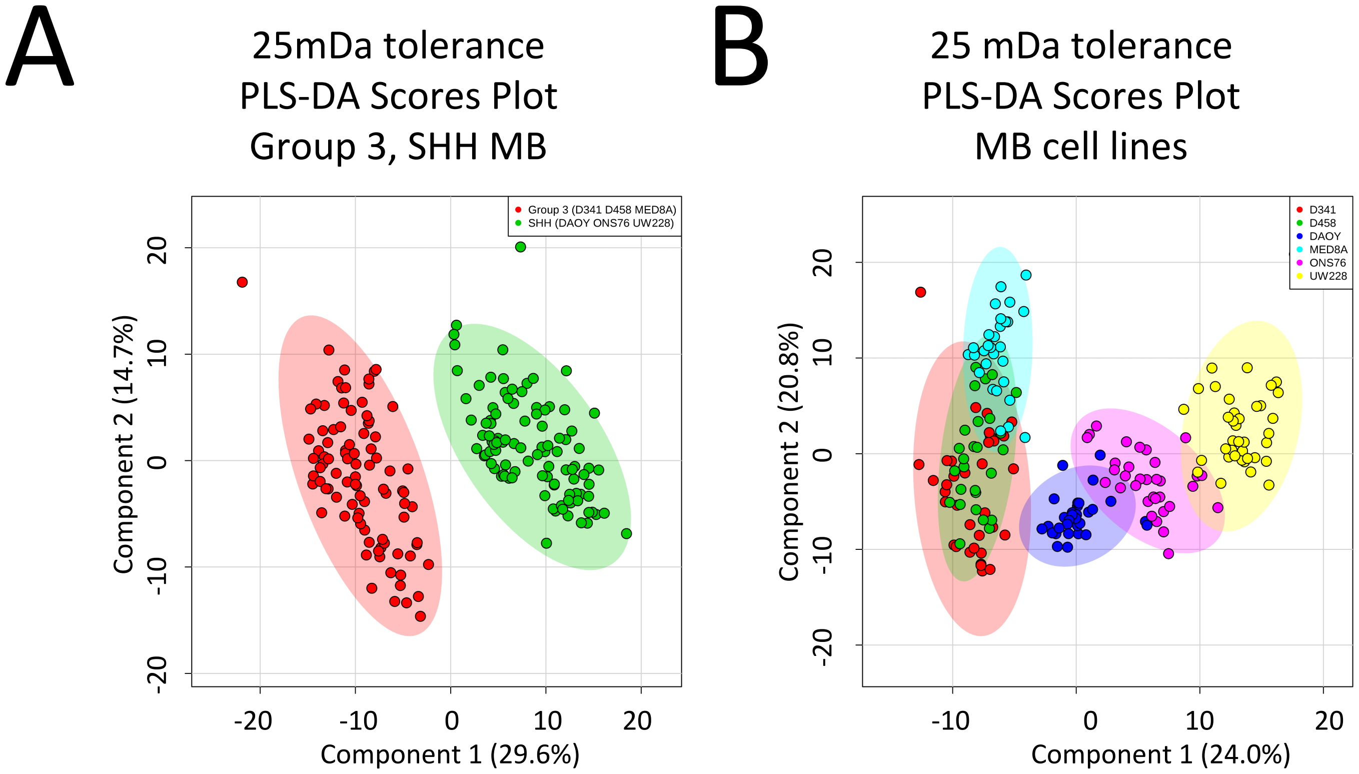

Figure S5. Statistical discrimination of MB subgroups and cell lines using mass corrected PIRL MS spectra. Here we used post processing to correct for mass shift in each dataset using internal mass lock compounds as described in the experimental methods to narrow the tolerance range to 25mDa. As can be seen same order of separation between Group 3 and SHH MB (A) and MB cell lines (B) are seen compared to results presented in Fig 2,3 that used a wider 100mDa mass tolerance range in the absence of individual dataset correction to account for day to day drift in mass shift due to instrument performance. See the next section "Analytical performance and the duty cycle" for more information.

Analytical performance and the duty cycle

Origin of outlier data points

The PIRL-MS spectra of two of the 3 outliers noted in Fig. 2A (UW228 sample E1 and ONS76 sample C8) possessed 140 and 170 mass peaks, respectively, upon application of a 5% noise level threshold. While this constitutes 35-45% fewer mass peaks compared to the average of 265±70 mass peaks for data points within the confidence interval, the high standard deviation seen in the number of mass peaks across the dataset makes this drop insignificant. In a similar vein, in the PIRL-MS spectrum of the third outlier (ONS76, sample B9) 250 mass peaks could be identified, which is comparable to the average value of all data points. Likewise, examination of MS signal strength using Total Ion Count (TIC) values suggested that outliers showed a maximum of only 2 fold drop from the average TIC value of $(1.5\pm0.9)\times10^6$ exhibited by the grouped data points. A comparison with desorption site heterogeneity from histology could not be made (given project design) to determine whether intrinsic sample heterogeneity (blood vessels, nerves etc) at the desorption site could have been responsible for the outlier behavior given they possessed good quality MS spectra. This comparison will be part of the workflow implemented for our future work on human samples, where greater heterogeneity is expected. Due to the ablative nature of our rapid tumour grading platform, histology only at the vicinity of the laser desorption sites can be accessed with post PIRL-MS staining and microscopy assessment. This creates a nontrivial level of uncertainty that could only be resolved by showing observations hold over a large number of patient samples. The TIC and the number of expected mass peaks typical for MB samples determined above, however, may be further utilized to establish and implement analytic criteria for acceptable PIRL-MS data quality on the basis of TIC threshold, intensity of most abundant expected peaks, or the number of mass peaks. This information will provide data inclusion/exclusion rules for single PIRL-MS events in an unbiased manner prior to commencing statistical modeling. On the basis of this information, one PIRL-MS data point that only contained 16 mass peaks was excluded from the analysis.

Duty cycle

We now discuss the analytic reproducibility of the PIRL-MS platform described in Fig. S1. With the PIRL laser providing a minimum of ~250 mW of average power, close to 90% of all of the PIRL-MS sampling attempts resulted in acceptable MS spectra in which greater than 100 mass peaks could be detected, after the application of a 5% noise threshold. The most prominent causes for the 10% failure rate in detecting MS signal were (1) laser tip contamination, (2) sample dehydration that resulted in tissue burning as opposed to desorption affecting the signal level, and (3) irreproducible plume capture due to flexible collection geometry and the weak suction at the proximal tip of the 2 m long collection tube (Fig. S1). Cleaning the laser tip by dipping it in methanol for 5 seconds in between sampling events significantly improved the duty cycle. In the absence of a Venturi pump akin to that implemented in REIMS[5, 6] (or recently with other infrared laser desorption systems[7]) to increase efficiency of the laser plume collection, we optimized the capture of the desorption plume by manually rotating the proximal tip of the flexible 2 m collection tube held 1-2 mm from the laser fiber tip at a 90 degree angle around the laser tip (Fig. S1). This allowed the operator to maximize the collection of the desorption plume, which is visible to the naked eye. This degree of freedom gave stronger, more reproducible signal compared to a fixed geometry that was also attempted (Fig. S1). To circumvent this shortcoming without integration with REIMS interface, a future aim of our technology development, the MS operator monitored the PIRL-MS signal strength and the quality of PIRL-MS spectra available in real time through our TOF instrument during the course of the 10-second sampling. The MS operator then guided the laser operator to continue sampling or whether to stop after the first 5 seconds of data

collection. Nevertheless, no data point was collected in excess of 10 seconds of sampling. Further, to provide realistic measures of performance no data point was taken out of the pool of the attempted PIRL-MS shots on any other grounds to skew the assessment of the duty cycle, except for one data point that contained only 16 mass peaks. We anticipate that the future integration with REIMS interface that offers Venturi action will aid greatly with the signal stability, and potentially reduce sampling time and area, leading to an improvement of the duty cycle of the PIRL-MS method. Including the spectral inclusion/exclusion criteria discussed above will allow automated assessment of the unacceptable, poor quality PIRL-MS data points prior to statistical analysis, reducing the chance of sampling error that could potentially lead to misclassification of tumour subgroup affiliation which may have grave consequences in a clinical setting.

Mass shift correction

For real time applications, while on-the-fly correction of mass shift is possible with REIMS using an external mass lock compound infused into the aerosol stream, to account for the day-to-day variations in instrument performance drift with our PIRL-MS setup we deliberately used a wide 100 mDa mass tolerance range for our multivariate analyses. However, to ensure that such a large mass tolerance window did not result in erroneous inclusion of mass peaks harboring the noise in our statistical assessment, we further showed that PIRL-MS datasets corrected for mass shift post processing resulted in essentially the same statistical separation with a narrower window of 25 mDa mass tolerance (Fig. S5). To achieve this low tolerance, we used internal mass lock correction post processing as detailed in the experimental section.

References

1. M. Woolman, A. Gribble, E. Bluemke, J. Zou, M. Ventura, N. Bernards, M. Wu, H. J. Ginsberg, S. Das, A. Vitkin and A. Zarrine-Afsar, *Scientific reports*, 2017, **7**, 468.
2. J. Xia, R. Mandal, I. V. Sinelnikov, D. Broadhurst and D. S. Wishart, *Nucleic acids research*, 2012, **40**, W127-133.
3. J. Xia, I. V. Sinelnikov, B. Han and D. S. Wishart, *Nucleic acids research*, 2015, **43**, W251-257.
4. J. Xia, N. Psychogios, N. Young and D. S. Wishart, *Nucleic acids research*, 2009, **37**, W652-660.
5. J. Balog, T. Szaniszlo, K. C. Schaefer, J. Denes, A. Lopata, L. Godorhazy, D. Szalay, L. Balogh, L. Sasi-Szabo, M. Toth and Z. Takats, *Anal Chem*, 2010, **82**, 7343-7350.
6. J. Balog, L. Sasi-Szabo, J. Kinross, M. R. Lewis, L. J. Muirhead, K. Veselkov, R. Mirnezami, B. Dezso, L. Damjanovich, A. Darzi, J. K. Nicholson and Z. Takats, *Sci Transl Med*, 2013, **5**, 194ra193.
7. B. Fatou, P. Saudemont, E. Leblanc, D. Vinatier, V. Mesdag, M. Wisztorski, C. Focsa, M. Salzet, M. Ziskind and I. Fournier, *Scientific reports*, 2016, **6**, 25919.
